# Dynamics, diversity, and roles of bacterial transmission modes during the first asexual life stages of the freshwater sponge *Spongilla lacustris*

**DOI:** 10.1186/s40793-024-00580-7

**Published:** 2024-06-08

**Authors:** Benoit Paix, Elodie van der Valk, Nicole J. de Voogd

**Affiliations:** 1 Naturalis Biodiversity Center, Leiden, The Netherlands; 2 UMR CARRTEL, INRAE - Université Savoie Mont-Blanc, Thonon-les-Bains, France; 3 Institute of Biology (IBL), Leiden University, PO Box 9505, Leiden, 2333BE The Netherlands

**Keywords:** Freshwater sponge, Holobiont, Microbiome, Vertical transmission, Horizontal acquisition, Gemmule, Ontogeny

## Abstract

**Background:**

Sponge-associated bacteria play important roles in the physiology of their host, whose recruitment processes are crucial to maintain symbiotic associations. However, the acquisition of bacterial communities within freshwater sponges is still under explored. *Spongilla lacustris* is a model sponge widely distributed in European rivers and lakes, producing dormant cysts (named gemmules) for their asexual reproduction, before winter. Through an in vitro experiment, this study aims to describe the dynamics of bacterial communities and their transmission modes following the hatching of these gemmules.

**Results:**

An overall change of bacterial *β*-diversity was observed through the ontology of the juvenile sponges. These temporal differences were potentially linked, first to the osculum acquisition and the development of a canal system, and then, the increasing colonization of the *Chlorella*-like photosymbionts. Gemmules hatching with a sterilized surface were found to have a more dispersed and less diverse microbiome, revealing the importance of gemmule epibacteria for the whole holobiont stability. These epibacteria were suggested to be vertically transmitted from the maternal tissues to the gemmule surface. Vertical transmission through the incorporation of bacterial communities inside of the gemmule, was also found as a dominant transmission mode, especially with the nitrogen fixers *Terasakiellaceae*. Finally, we showed that almost no ASVs were shared between the free-living community and the juveniles, suggesting that horizontal recruitment is unlikely to happen during the first stages of development. However, the free-living bacteria filtered are probably used as a source of nutrients, allowing an enrichment of copiotrophic bacteria already present within its microbiome.

**Conclusions:**

This study brings new insight for a better understanding of the microbiome acquisition during the first stages of freshwater sponge development. We showed the importance of epibacterial communities on gemmules for the whole holobiont stability, and demonstrated the near absence of recruitment of free-living bacteria during the first stages.

**Supplementary Information:**

The online version contains supplementary material available at 10.1186/s40793-024-00580-7.

## Introduction

Sponges (phylum Porifera) are filter-feeding and sessile animals known as important ecosystems engineers in benthic environments. Among their essential roles, they participate to the cycling of the organic matter, and provides a habitat by hosting a large diversity of micro- and macro-organisms [1]. Described as a functional entity, the tight association of a sponge and its microbiome is called “sponge-holobiont”. Within these holobionts, microbial symbionts can displays key functions to the host, by producing chemical defenses or supplying additional nutrients, among others [2]. Sponge microbiomes are often described to be diverse, stable and host-specific [3], playing an essential role for the resilience of their host observed during environmental changes (e.g. under heat-stress or acidification [4, 5]). The mechanisms associated with the selection of microbial symbionts and their stable maintenance are increasingly studied for marine sponge models [6, 7]. Among others, stochasticity processes seem to be involved through the acquisition of microbial communities [8, 9]. The current body of knowledge suggests that marine sponges can acquire their microbial communities through both vertical and horizontal transmission (“VT” and “HT”, respectively; [7, 9–11]). The intermediate transmission mode was initially named “leaky vertical transmission” [12], or “mixed-mode transmission” [13]. While VT was confirmed for a large diversity of sponge models [14–18], it appears insufficient to explain the origin of the overall bacterial community [8, 19, 20]. For example, larvae of eight Mediterranean sponge species shared only 17% of the bacterial ASVs with their parents, on average [8]. Additionally, the importance of horizontal transmission was discussed for 19 sponge species from Vietnam, as their bacterial core community was found to be highly shared (> 50%) with the core bacterial community of the ambient seawater [19]. In this latter study, species-specific recognition mechanisms were hypothesized for the sponges, allowing an enrichment of specific planktonic bacteria. However, the diversity and relative importance of these transmission modes are still unknown within freshwater sponges, as their bacterial diversity remains largely underexplored [21].

Freshwater sponges (Order Spongillida) comprise approximately 240 species [22], and display a wide range of environmental adaptation within diverse ecosystems such as lakes, rivers, streams, ponds, or urban canals [23]. They can survive drastic changes of temperatures and light, but also desiccation and anoxic conditions, and can also tolerate high levels of pollutants [23]. Under light-exposed conditions, the establishment of stable interaction with photosymbionts (e.g. microalgae such as *Chlorella* spp.) constitutes an important factor for the physiology of freshwater sponges harboring a green coloration [24]. Up to 20% of the photosynthates produced by the microalgal partners can be translocated directly to the host [25, 26]. However, symbiotic association within freshwater sponges can be disturbed, leading for example to a dysbiosis state of the baikal sponge *Lubomirskia baikalensis* harboring the brown rot syndrome associated with bleached tissues [27]. Through genetic regulations, the host immunity response can also be involved in the recognition of symbiotic association, as demonstrated through the infection of aposymbiotic sponge *Ephydatia muelleri* with their *Chlorella*-like algal symbionts [28]. These results revealed the importance of such symbionts for the host, and suggested that they can also be horizontally acquired [28], in addition to the vertical transmission [29]. However, the mechanisms associated with the recognition and the selection of a host-specific bacterial community from their environment are yet to be explored for freshwater sponges [30]. Indeed, the bacterial composition has been rarely compared between freshwater sponge species, but also with those from the ambient environment (e.g. from freshwater or sediment [21]). Sugden et al. showed that bacterial communities of *E. muelleri* are mainly distinct from those from the surrounding freshwater and biofilms [31]. Geographical differences were also observed between rivers, suggesting that the environment could also shape the host-microbe specificity. In this latter study, horizontal acquisition from ambient bacteria was suggested as an important factor that could explain the rivers-specific microbiome, while the relative importance of the vertical transmission was still uncertain.

*Spongilla lacustris* (Linnaeus, 1759) is one of the most widespread freshwater sponge species in temperate areas of the Northern Hemisphere [32]. As for *E. muelleri*, the asexual reproduction of the *S. lacustris* model has been extensively described through the production (in autumn) of diapausing cysts, named gemmules [33]. These gemmules contain undifferentiated dormant stem cells (named thesocytes) and provide protection against adverse conditions during winter or exposure to air. These gemmules can be hatched and cultivated under diverse in vitro conditions (allowing a detailed description of their first developmental stage). Hence, gemmule-producing sponges constitute models of interest for recent imaging and molecular techniques [28, 34–36]. However, the microbiome composition of *S. lacustris* has been underexplored to date, in regards to their wide distribution area. Only two studies have been conducted on its microbiome, both relying on culture-dependent approaches [37, 38]. These studies suggested the dominance of Alphaproteobacteria, Actinobacteria, Bacteroidota, Gammaproteobacteria, and Betaproteobacteria [37, 38]. The mechanisms underlying the early assembly of the overall bacterial consortium within these freshwater sponges are yet to be explored.

Our study aims to decipher the transmission modes of bacterial communities during the first steps of *S. lacustris* asexual cycle. We hypothesize that the mixed mode transmission occurs, with both VT and HT. First, the VT would be achieved through the transmission of maternal bacteria within the gemmule, but also on its surface, as suggested by Sugden et al. [31]. Once the gemmule has hatched and the filtration system is formed, the HT of planktonic bacteria could then constitute an additional factor shaping the *S. lacustris* microbiome. To test these hypotheses, an experimental design is developed to determine the respective importance over time of (i) the microbiome transmitted on the gemmule’s surface, and (ii) the ambient free-living bacteria filtered by the juveniles.

## Materials and methods

**Fig. 1:**
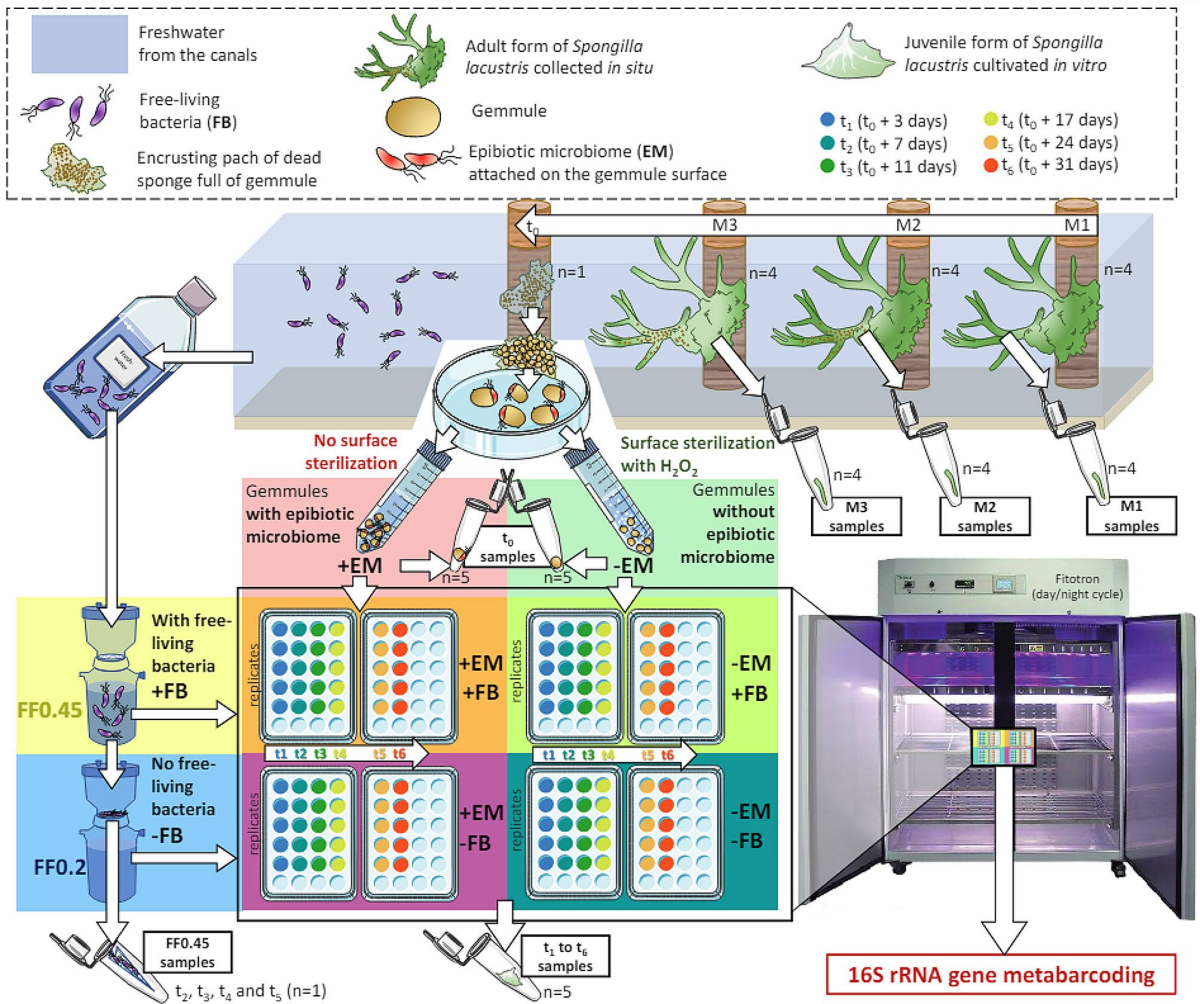
Workflow summarizing the experimental design of the study. *Abbreviations* EM for epibiotic microbiome, and FB for free-living bacteria

The overall study design was based on the cultivation of the *S. lacustris* juveniles under two crossed factors (resulting in 4 treatments, Fig. 1). The first factor was related to the absence/presence of epibiotic microbiome (± “EM”) on the gemmule, depending on the surface sterilization. In parallel, the second factor was related to the absence/presence of free-living bacteria (± “FB”) in the cultivation medium, depending on the pore size of the filters used for the freshwater filtration.

### Sampling of the in situ adult sponges

Four replicates of adult *S. lacustris* specimens were collected in situ from a wooden jetty located at an inlet of the river Rhine at Oegstgeest (the Netherlands, 52°10’24.5"N, 4°27’01.7"E, ~ 0.5 m depth), at three different sampling times a month apart in 2021: M1 (October 15th), M2 (November 10th) and M3 (December 15th). Sponge samples were rinsed with surrounding freshwater filtered upon 0.2 μm pore size filters (FF0.2), and cut with sterilized tweezers and scalpels. The identification of *S. lacustris* was based on skeletal and gemmoscleres examination. A voucher specimen is deposited at the sponge collection of Naturalis Biodiversity Center (RMNH.POR.12,472). Samples for DNA metabarcoding were preserved in sterilized plastic vials filled with 96% ethanol and conserved at -20 °C until DNA extraction. After M3, only encrusting parts of dead sponges were left: the sponge skeletons were observed without tissues, but full of gemmules. One of these “encrusting gemmules patches” (resulting from specimens collected at M3) was collected at t_0_ (February 17th) in sterilized plastic vials filled with surrounding FF0.2. These samples collected for the experiment were kept at 4 °C until the washing steps of the gemmules.

### Sampling and washing of the gemmules

One day after the sampling, gemmules from the encrusting patch were separated from the maternal sponge skeleton according to [34], using sterilized tweezers and teasing needles. Approximately 450 gemmules were evenly distributed in two distinct 15mL falcon tubes. Gemmules from the first tube were washed according to [34], while gemmules from the second tube were washed with the same protocol but with Strekal’s medium instead of the 1% hydrogen peroxide (H_2_O_2_) solution (Fig. 1). A 10X solution of Strekal’s medium was prepared with 0.9mM of MgSO_4_-7H_2_O, 0.5mM CaCO_3_, 0.1mM Na_2_SiO_3_-9H_2_O and 0.1mM KCl. For this treatment without H_2_O_2_, particular cautions were taken (using a stereomicroscope) to sort and remove the dead and damaged gemmules, as well as important debris of the sponge maternal skeleton attached to the gemmule surface. As the H_2_O_2_ solution induces the sterilization of the gemmule surfaces [34], these two washing conditions allowed to separate the gemmules in two groups: (i) the -EM for the sterilized gemmules without any potential epibiotic microbiome, and (ii) the + EM for the unsterilized gemmules with their potential epibiotic microbiome still attached the surface (Fig. 1). Gemmules were stored at 4 °C in Strekal’s medium for one hour before the plating. The efficiency of the sterilization through the use of H_2_O_2_ was investigated as described in Supplementary Information (SI).

### Plating and cultivation of the juveniles

For the t_0_ samples (gemmules collected before the plating), 5 replicates for both + EM and -EM treatments were collected from the 15mL falcon tubes and stored in 1.5mL eppendorf tubes filled with 96% EtOH at -20 °C until DNA extraction. A total of 120 juvenile samples were expected for the experiment, based on the calculation of 6 sampling times (t_1_ to t_6_), 2 conditions of gemmule surface (± EM), 2 conditions of filtered freshwater (± FB), and 5 replicates.

Under sterile conditions, the + EM and -EM gemmules were distributed (on ice) in 120 wells of 24-well plates (approximately 3 to 4 gemmules per well), as described in Fig. 1. The Strekal’s medium was then removed and replaced by 1 mL of filtered freshwater at room temperature. Plates were filled with freshwater filtered upon 0.45 μm size filters (FF0.45) or FF0.2, for + FB and -FB respectively (Fig. 1). The filtered freshwater used was collected 1 h before the filtration, on the same sampling site as the in situ sponges. The plates were then transferred in an incubation chamber (Fitotron®, Weiss Technik, GmbH, Germany) at 20 °C, with 70% of humidity and a day/night cycle of 14:10 h (960 lx for the day light, SD: ±65).

Details of the sampling timeline of the experiment are described in **note S1** (Supplementary Information, SI). For each treatment of each sampling time (t_1_ to t_6_,), the hatching rates obtained (80.2% in average, SD: ± 21.2%) allowed us to collect at least one hatched juvenile for each replicates. The unhatched gemmules were discarded, resulting in approximately 1 to 3 juveniles gathered per replicate. Briefly, samples from t_1_ to t_6_ were collected at 3, 7, 11, 17, 24, and 31 days after the plating (t_0_), respectively. The experiment was designed up to 31 days to follow potential changes occurring after the formation of the osculum and the development of a mature canal system. For each sampling time, the replicates of juvenile sponges were collected in 1.5mL eppendorf tubes filled with 96% EtOH at -20 °C until DNA extraction.

The FF0.45 and FF0.2 were refreshed at room temperature every 3 or 4 days with freshly collected freshwater from the same sampling site described above (see details in SI, **note S1**). This duration between two refreshments was slightly longer compared to those recommended in [34] (every 2 days). This choice was considered to have a longer exposure time enabling potential recognition mechanisms allowing the horizontal acquisition of planktonic bacteria.

To investigate the structure of the free-living bacterial community amended at each refreshment for the + FB condition, the FF0.45 was also successively filtered upon 0.2 μm pore size PES filters used for DNA extraction. These samples named “FF0.45 samples” were collected for refreshment occurring before each time (except t_1_), and preserved in the CTAB extraction buffer at -20 °C until extraction.

Before each sampling time from t_1_ to t_6_, the juveniles sponges from each treatment (cross-conditions of ± EM with ± FB) were photographed using a stereomicroscope (SteREO Discovery.V20, ZEISS, Germany) mounted with a camera (AxioCam MRc5, ZEISS, Germany).

### DNA extractions, library preparation and high throughput sequencing of 16S rRNA gene amplicons

DNA from replicates of in situ adults (M1, M2, and M3), gemmules (t_0_), and in vitro juveniles (t_1_ to t_6_) were extracted using the FastDNA™ SPIN Kit for Soil (MP Biomedicals, Inc.) following the manufacturer’s instructions. In situ adult sponge samples were cut into small pieces of approximately 3*1*0.5 mm using sterilized tweezers and scalpel blades. Special care was taken to collect only sponge tissues, avoiding gemmules. For t_0_ samples, the gemmule coating was broken (by crushing them using the top of 10µL filter pipette tips), to facilitate the extraction of the DNA from the inside of the gemmule (see **note S2** in SI). The CTAB DNA extraction of the FF0.45 samples was performed as described in [39].

The library preparation was conducted through a two-step PCR protocol for all samples together with the extraction blank and two negative controls (mQ water instead of template DNA). For the first PCR, the V3-V4 regions of the 16S rRNA gene were targeted and amplified with the PCR primers 341F 5’-CCTACGGGNGGCWGCAG-3’ and 785R 5’-GACTACHVGGGTATCTAATCC-3’ [40] and the KAPA HiFi HotStart Ready Mix PCR Kit (Roche Molecular Systems, Inc.). Reactions were performed in a T100 Thermal Cycler (Bio-Rad, Hercules, CA, United States). The following thermal cycling scheme was conducted: initial denaturation at 95 °C for 3 min, 30 cycles of denaturation at 98 °C for 20 s, annealing at 57 °C for 30 s, followed by extension at 72 °C for 30 s. The final extension was carried out at 72 °C for 1 min.

PCR products from the samples were checked using an E-Gel™ (agarose gels at 2%), and the absence of amplification was validated for the negative controls and two extraction blanks. In several gemmule and juvenile samples, PCR amplifications were unsuccessful due to low DNA concentrations. At least 4 replicates was successful for every treatment at each time, except for + EM + FB at t_5_, with 3 successful replicates (Table S1). PCR products were then cleaned with NucleoMag NGS-Beads (bead volume at 0.9 times the total volume of the sample, Macherey Nagel, Düren, Germany) using the VP 407AM-N 96 Pin Magnetic Bead Extractor stamp (V&P Scientific, San Diego, CA, United States). Through a second PCR, 3 µL of the cleaned PCR products were then amplified and labeled using the MiSeq Nextera XTDNA library preparation kit (Illumina, San Diego, CA, United States), with the same thermal cycling scheme limited to 8 cycles. PCR products were then analyzed with the Fragment Analyzer Agilent 5300 using the DNF-910-33 dsDNA Reagent Kit (35–1,500 bp) protocol (Agilent Technologies, Santa Clara, CA, United States) to confirm the successful labeling of the DNA fragments. Negative controls and extraction blanks remained negative after this step. The pooling at the equimolar concentration was performed with QIAgility (Qiagen, Hilden, Germany). The final pool was then cleaned with NucleoMag NGSBeads, eluted in Milli-Q, and the DNA concentration was verified using Tapestation 4150 (Kit HSD 5000, Agilent Technologies, Santa Clara, CA, United States). The sequencing was performed on an Illumina MiSeq V3 PE300 platform at BaseClear B.V. (Leiden, Netherlands).

### 16S rRNA gene metabarcoding data processing

The raw reads were first treated by BaseClear B.V. for demultiplexing (using bcl2fastq version 2.20, Illumina), and filtering based on two quality controls (using Illumina Chastity filtering, and a PhiX control signal filtering). The following reads were then processed with the DADA2 workflow allowing an inference to Amplicon Sequence Variant (ASV) [41, 42], using the “dada2” R package following the workflow described in [43] and guidelines described in the online tutorial (https://benjjneb.github.io/dada2/tutorial.html). The parameters used for filtering and trimming reads were as follows: truncation length of 270 and 240 base pairs for forward and reverse reads, respectively, maxN = 0, maxEE = 2, and truncQ = 2. After the construction of the ASV table, chimeric sequences were filtered and the taxonomic assignment was performed using the Silva v138 reference database [44].

The ASV and taxonomy tables produced by the pipeline were then combined into a phyloseq object, together with the sample metadata table, using the “phyloseq” R package [45]. The dataset was then filtered by removing all sequences from Eukaryota, chloroplast and mitochondria (representing on average > 0.01% [SD: +/-0.002], 36.9% [SD: +/-12.8] and 0.9% [SD: +/-1.9] of all reads per sample, respectively). Data was then decontaminated with the negative controls and the two extraction blanks used as control samples, using the “decontam” R package [46].

### Metabarcoding and statistical analyses

The *α*-diversity measures were estimated with Chao1 (estimated richness), Pielou (evenness), and Shannon (both richness and evenness) indices using the “phyloseq” and “vegan” R packages [45, 47] and the rarefied datasets (rarefaction performed to the minimum library size, i.e. 5332 reads). According to Shapiro tests, the diversity metrics were significantly different from the normal distribution. Consequently, differences between sample groups within these metrics were investigated through non-parametric tests (Kruskal-Wallis followed by pairwise Wilcoxon tests) using the “agricolae” R package [48]. Following recommendations for compositional approaches from [49, 50], all other analyses were conducted without rarefaction, using the “phyloseq” R package and the datasets normalized to the total number of sequences per sample. The *β*-diversity was analyzed with non-metric multidimensional scaling (NMDS) using Bray-Curtis dissimilarity. Differences of *β*-diversity between groups were statistically checked with one-way permutational multivariate analysis of variance (PERMANOVA) tests followed by pairwise Adonis tests, using the “vegan” R package. Differences between the dispersion of the *β*-diversity within each treatment were calculated through the multivariate dispersion (PERMDISP) analysis using the *betadisper*() function from the “vegan’ R package, and tested using an analysis of variance (ANOVA) followed by a Tukey’s HSD pairwise test.

Focusing on t_0_ and t_1_ samples, phylogenetical heat trees were performed to identify the significant pioneer taxa differentially abundant between the different treatment conditions (± EM and ± FB), using the *metacoder* package [51]. The differential analyses were performed with a subset of the compositional dataset excluding rare ASV (relative abundance < 0.02%).

Analyses of the ASVs shared between conditions were performed with the *core*() function using the “microbiome” R package [52]. An ASV from juveniles from one specific treatment at a specific sampling time was considered shared with the in situ adult sponges, when the ASV is present at least in 3 replicates of each of the two groups. Similarly, an ASV from juveniles from one specific treatment at a specific sampling time was considered shared with the filtered freshwater (FF0.45), when the ASV is present at least in 3 samples of each of the two groups. Using the “eulerr” R package [53], the Venn diagrams were plotted to represent the number of shared ASVs between the juveniles, the in situ adult sponges and the prefiltered freshwater with the *venn*() function.

## Results

### Development of the juvenile sponges

The hatching of the gemmules was observed 2 days after their plating (t_0_), whatever the treatment. No significant differences of hatching rates were observed between the gemmules from each treatment (ANOVA test: *p* = 0.07). These hatching rates reached in average 76.3% (SD: ±20.7) for + EM + FB, 81.9% (SD: ±22.6) for + EM-FB, 75.3% (SD: ±21.4) for -EM + FB and 87.2% (SD: ±20.0) for -EM-FB. The same developmental stages were observed between treatments for each sampling time (Figure S1). The first sampling time (t_1_) was characterized with the development of the sponge tissues on the gemmule coat (i.e. the gemmule husk) and colonizing the substrate. The early formation of a canal system and an osculum was observed for t_2_ samples. At t_3_, the development of a mature canal system was noticed, together with a light green coloration. The coloration was more saturated at t_4_ and thereafter (Figure S1).

### Alpha-diversity analyses

**Fig. 2:**
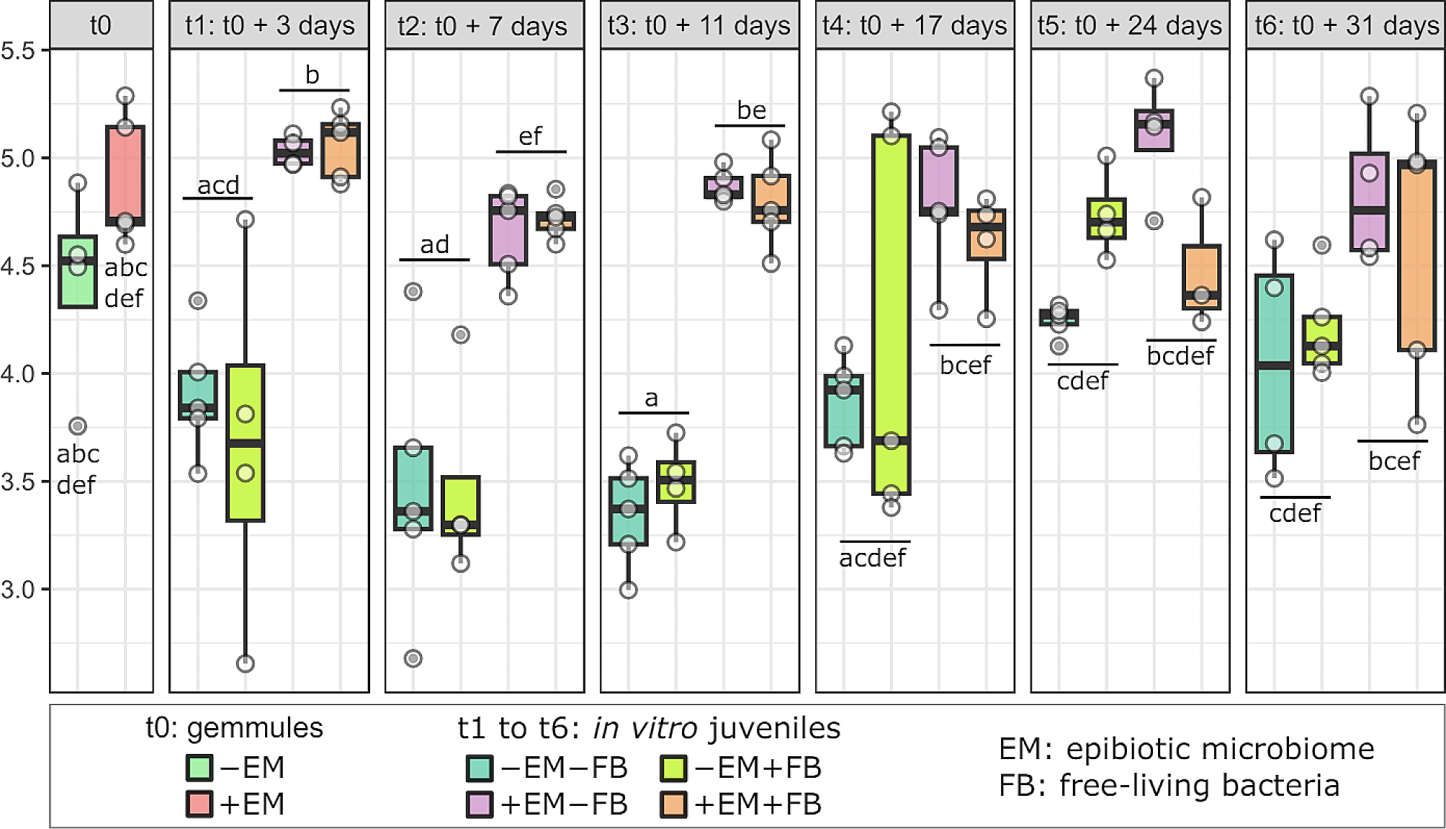
Dynamics of the *α*-diversity (Shannon index) of the bacterial communities associated with the gemmules (t_0_) and the in vitro juvenile sponges (t_1_ to t_6_). Lowercase indices (from a to f) indicate the results from the Wilcoxon pairwise tests comparing ± EM samples within each time. *Abbreviations* EM for epibiotic microbiome, and FB for free-living bacteria

Significant differences between the different sample types were observed only for the Pielou index (Figure S2, Table S2), with lower values within in situ adult sponges compared to gemmules and juvenile sponges (Figure S2, Wilcoxon test). For gemmules and juveniles, significant differences between treatments were observed according to the sampling time together with the ± EM (with epibiotic microbiome) factor, for the Shannon (Fig. 2, Table S3), Chao1 and Pielou indices (Figures S3A and S3B, respectively, Table S3). No significant differences were observed according to the ± FB factor (Kruskal-Wallis test: *p* > 0.05 for the three indices). For the Shannon index, a decrease from t_0_ to t_2_ was observed for the -EM (without epibiotic microbiome) samples (Fig. 2). At t_1_, t_2_, and t_3_, Shannon values in -EM samples were significantly lower compared to those in + EM (Fig. 2, Wilcoxon test). The same results were observed for the two other indices (Pielou and Chao1), except at t_2_ for the Chao1 (Figure S3, Wilcoxon test). For all indices, a general tendency of increasing *α*-diversity was observed after t_3_ up to t_6_, for the juveniles in -EM samples, while the values for the + EM samples stayed stable all along the experiment.

### Beta-diversity analyses

**Fig. 3:**
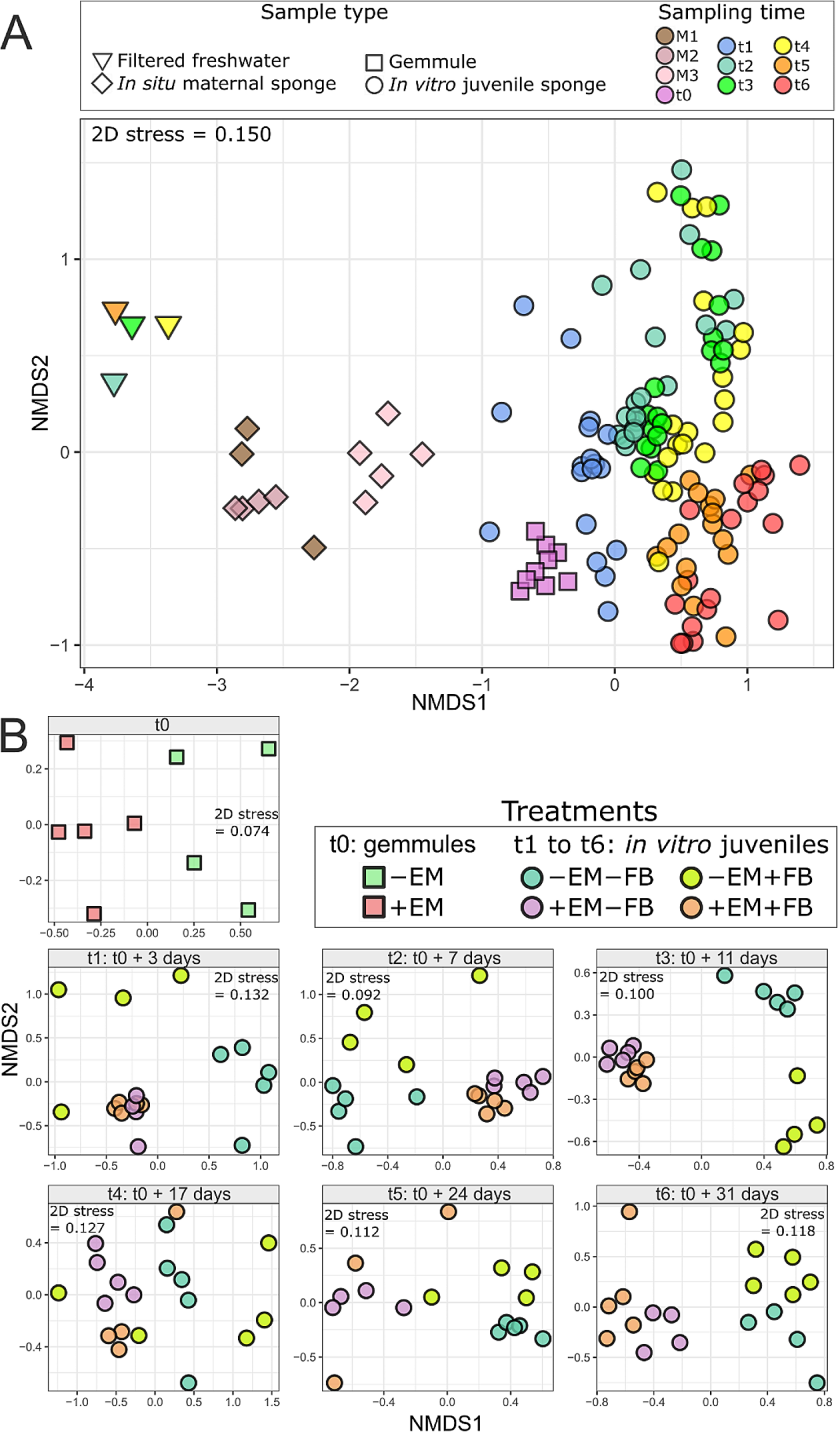
*β*-diversity of the bacterial communities (Bray-Curtis distances). **A** NMDS plot performed with all samples represented according to their sample type and sampling time. **B** NMDS plots performed for each sampling time of the in vitro juveniles, separately, with samples represented according to their treatments. *Abbreviations* EM for epibiotic microbiome, and FB for free-living bacteria

The NMDS plot and PERMANOVA test, conducted with all samples, showed significant differences between the *β*-diversity of each sample type (freshwater, in situ adult tissues, gemmules and in vitro juveniles, Fig. 3A, Table S4). Additionally, significant temporal changes were observed on the NMDS along (i) the first axis from M3 to t_0_ (in situ adult sponges and gemmules), and (ii) the second axes from t_1_ up to t_6_ (in vitro juveniles) (Fig. 3A; Table 5 A). Despite a clear temporal trend of the *β*-diversity within in vitro juveniles, the pairwise comparison tests revealed that most temporal shifts from one sampling time to the next were not significant, except between (i) t_1_ and t_2_, and (ii) t_4_ and t_5_ (Fig. 3A, Table S5B). For in vitro juvenile samples, the three-way PERMANOVA (Table S5A) also indicated significant differences between sampling times, in combination with the two treatment factors (± EM and ± FB). Pairwise comparison tests conducted with all juvenile samples, showed an overall significant difference between the *β*-diversity the 4 treatments, except between + EM + FB and + EM-FB (Table S5C). The NMDS of all samples plotted with a color code related to the treatments (Figure S4) revealed that + EM samples from t_1_ to t_4_ are grouped, while differences between + FB and -FB can be distinguished for the -EM samples. Additionally, differences between + EM and -EM can also be observed with t_5_ and t_6_ samples (Figure S4).

The NMDS (Fig. 3B**)** and two-way PERMANOVA (Table S6A) analyses, performed at each sampling time separately, revealed significant differences of *β*-diversity between treatments. At t_0_ (for the gemmules), significant differences between + EM and -EM samples were observed on the first NMDS axis (Fig. 3B, Tables S6A and S6B). For the NMDS plots from t_1_ to t_6_, significant differences can also be observed between juveniles + EM and -EM (Fig. 3B, Tables S6A and S6B). From t_1_ to t_3_, a similar pattern can be observed with three clusters differentiating (i) + EM samples (including both + and -FB), (ii) -EM + FB samples, (iii) and -EM-FB samples (Fig. 3B). More precisely, the differences between + FB and -FB appear more important within the -EM group than the + EM group. From t_4_ to t_6_, a larger separation between the + FB and -FB clusters was observed for the + EM samples. Finally at t_6_, 4 distinct clusters can be observed for each of the 4 treatments from the two cross-conditions: the first NMDS axis is involved in the ± EM differences, while the second axis seems to explain the ± FB differences. However, according to the pairwise comparisons, these ± FB differences at t_6_ were not significant, in both + EM and -EM conditions (Table S6B).

The *β*-diversity dispersion within each treatment (± EM and ± FB) was determined at each sampling time of the in vitro juveniles (Figure S5). A global tendency of increasing dispersion is observed with a higher dispersion over time for the + EM samples, confirming the previous observations on the NMDS plots (Fig. 3B). For -EM juvenile sponges, the highest dispersion values were found at t_1_, followed by a decrease at t_2_ (Figure S5). At t_1_, a significantly higher dispersion was observed in -EM compared to + EM, whatever the presence or not of FB (Figure S5, Table S7). At t_2_, a similar observation was made, with significantly higher dispersion in -EM compared to + EM, only in the + FB condition (Figure S5, Table S7). For t_3_, t_4_, and t_6_, no significant differences of *β*-diversity dispersion were observed between treatments (Figure S5). At t_5_, significant differences were observed with higher dispersion in + EM + FB juveniles compared to all -FB juveniles (Figure S5).

### Compositional and differential analyses

The composition of the bacterial community at the family level showed overall differences between each sample type (filtered freshwater, in situ adult sponge, the gemmules, and the in vitro juveniles, Figure S6). For the filtered freshwater, the main free-living bacterial communities were dominated by *Sporichthyaceae* and *Microbacteriaceae* (Actinobacteria), and *Burkholderiaceae* and *Methylophilaceae* (Gammaproteobacteria) (Figure S6A). For the in situ adult sponges, the composition was dominated by *Sporichthyaceae* (Actinobacteria), *Chitinophagaceae* and *Flavobacteriaceae* (Bacteroidia), *Elsteraceae* and *Terasakiellaceae* (Alphaproteobacteria) and *Comamonadaceae* (Gammaproteobacteria) (Figure S6A). Differences in composition for these adult sponge tissues were observed over time from M1 (October) to M3 (December), with a relative increase of *Flavobacteriaceae* and *Elsteraceae* and a relative decrease of *Sporichtyaceae*. Additionally, higher relative abundances of *Terasakiellaceae* can be observed specifically at M2 compared to M1 and M3.

For the gemmules (t_0_), the bacterial composition of the -EM was dominated by *Terasakiellaceae*, while the + EM was dominated by the *Rhodobacteraceae* and *Comamonadaceae* (Figure S6A). For the in vitro juveniles (Figure S6B), important changes can be observed, with a dominance of *Flavobacteriaceae* with exclusively ASVs from *Flavobacterium* (Bacteroidia;) at t_1_. From t_2_ to t_4_, *Comamonadaceae*, *Alteromonadaceae*, and *Pseudomonadaceae* (Gammaproteobacteria) are the dominant families. Alphaproteobacteria was also found as one of the most abundant classes (*Rhodobacteraceae*, *Sphingomonadaceae*, *Rhizobiaceae*), without clear changes observed over time. Additionally, an increase of *Pirellulaceae* can be noticed all along the experiment up to t_6_.

Differences in relative abundances of families between treatments were observed, for example at t_1_, with higher percentages of *Comamonadaceae* in + EM compared to -EM. For + EM at t_1_, higher abundances of *Oxalobacteraceae* were observed in + FB compared to -FB samples, while the opposite is noticed for *Alteromonadaceae*. At t_2_ higher relative abundances of *Pseudomonadaceae* were found in -EM samples compared to + EM samples, while the opposite was observed for the *Comamonadaceae*.

The differential analysis with t_0_ samples (gemmules before hatching) was performed to identify significant taxa differentially abundant in + EM gemmules compared to -EM gemmules, and conversely (Figure S7). The analysis confirmed observations from the barplots for the most abundant families (Figure S6A) with a higher abundance of *Terasakiellaceae* in the -EM, and a higher abundance of *Rhodobacteraceae* and *Comamonadaceae* + EM. These two last families were found mainly represented by the genus *Pseudorhodobacter* and *Hydrogenophaga*, respectively, being also found differentially more abundant in + EM samples. Other taxa with lower percentages were also found to be differentially abundant, such as *Burkholderiaceae* (genus *Ralstonia*) and *Hyphomonadaceae* (genus *Hirschia*) in + EM and *Chitinophagaceae* (genus *Ferruginibacter*) and *Pseudomonadaceae* (genus *Pseudomonas*) in -EM.

**Fig. 4:**
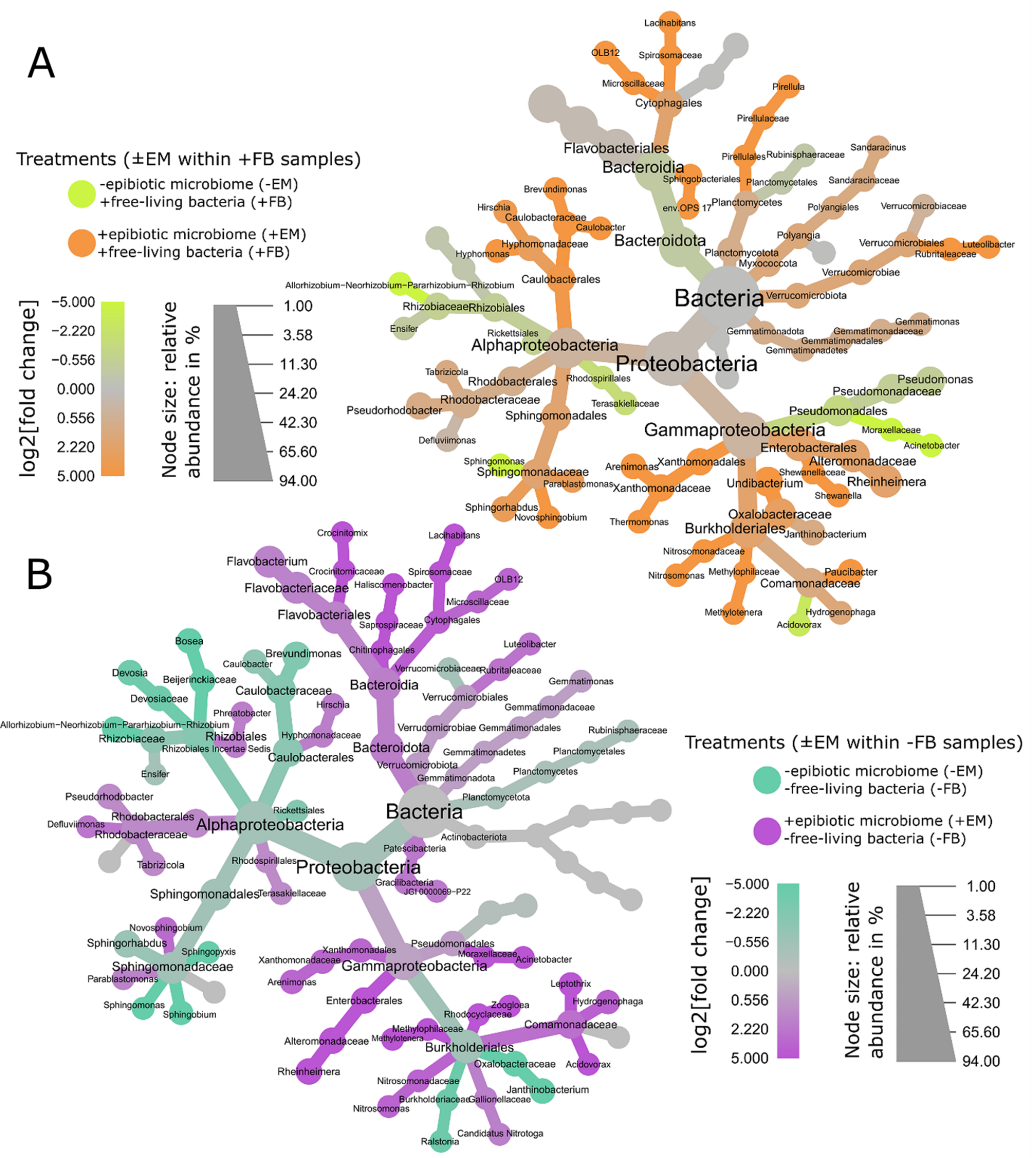
Phylogenetical heat trees performed with juvenile sponges samples collected at t_1_ and representing the taxa significantly and differentially abundant between the + EM and -EM treatments, within + FB (**A**) and -FB samples (**B**). For each taxon, (i) the colors of their associated nodes correspond to the log2 fold change between the ratio of the mean relative abundance within each treatment, (ii) the size of the nodes corresponds to the relative abundance of each taxon. *Abbreviations* EM for epibiotic microbiome, and FB for free-living bacteria

In addition to the t_0_ samples, another focus was made at t_1_ for differential analyses performed between treatment of the in vitro juveniles. The differential analysis between + EM and -EM was conducted for both + FB and -FB samples, separately. (i) For + FB samples, (Fig. 4A) significantly higher relative abundances of Caulobacterales and Sphingomonadales (Alphaproteobacteria), Cytophagales (Bacteroidia), Burkholderiales, Xanthomonadales and Enterobacterales (Gammaproteobacteria) were observed in + EM compared to -EM. Conversely, a significantly higher abundance of *Terasakiellaceae* (Alphaproteobacteria) and *Moraxellaceae* (Gammaproteobacteria) was observed in -EM compared to + EM. (ii) For -FB samples (Fig. 4B), significantly higher abundance of all Bacteroidia taxa (including Cytophagales), *Rhodobacteraceae* (Alphaproteobacteria), Enterobacterales and *Comamonadaceae* (Gammaproteobacteria) were observed in + EM compared to -EM, while higher abundance of *Oxalobacteraceae*, *Burkholderiaceae* and *Caulobacteraceae* and Rhizobiales were observed in -EM. The differential analysis between + FB and -FB was also conducted for both + EM and -EM samples, separately. (i) For + EM samples (Figure S8A), significant higher relative abundance of *Oxalobacteraceae* (genera *Undibacterium* and *Janthinobacterium*) and *Shewanellaceae* were observed for + FB samples compared to -FB samples, while *Moraxellaceae*, *Crocinitomicaceae*, *Alteromonadaceae* (*Rheinheimera*), *Rhodocyclaceae* where significantly more abundant in -FB samples compared to the + FB ones. (ii) For -EM samples (Figure S8B), the differential analysis revealed significant higher relative abundances of *Flavobacteriaceae* and Pseudomonadales (including *Moraxellaceae*) in + FB, while -FB samples were enriched with Caulobacterales, Rhizobiales, and *Sphingomonadaceae*.

### ASVs from gemmules or juveniles shared with adult sponges or freshwater samples

Venn diagrams (Figure S9) were generated to estimate the number of ASVs shared between three groups of samples: (i) the juveniles (from a specific treatment at a specific time), (ii) adult sponges and (iii) freshwater samples. Only three ASVs were found to be shared between the freshwater and the gemmules, or between the freshwater and the juveniles (Figure S9):(i) ASV73 (*Polynucleobacter*) shared at t_1_ and t_3_ with the + EM + FB juveniles, and at t_4_ with the -EM-FB juveniles, (ii) ASV80 (Candidatus *Limnoluna*) shared at t_5_ with the + EM + FB, -EM + FB and -EM-FB juveniles, and (iii) ASV734 (*Bradyrhizobium*) shared at t_6_ with the -EM-FB juveniles. These three ASVs (73, 80, and 734) were found with an average relative abundance below 0.04%. Additionally, a total of 17 ASVs were found to be shared between the freshwater and the in situ adult sponges (Figure S9).

**Fig. 5:**
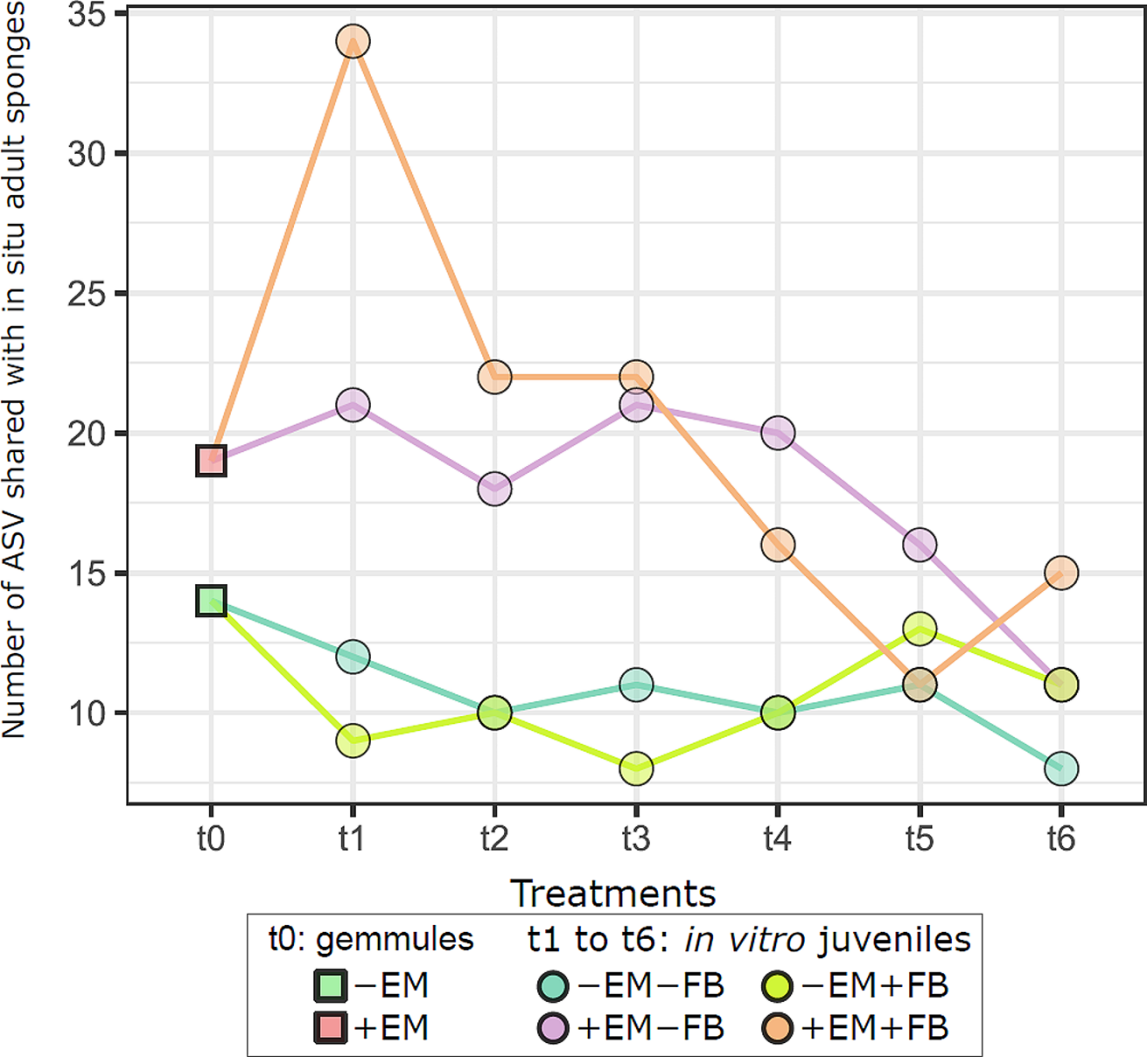
Dynamics of number of ASVs shared between gemmules/in vitro juveniles and in situ adult sponges. *Abbreviations* EM for epibiotic microbiome, and FB for free-living bacteria

Based on the Venn diagram results (Figure S9), the numbers ASVs shared between in vitro juveniles and in situ adult sponges were summarized in Fig. 5. These numbers were higher for + EM juveniles, compared to -EM juveniles. For example, at t_1_, 34 ASVs from the adult sponges were shared in + EM + FB, while only 9 were shared between the -EM + FB and the in situ adult sponges (Fig. 5 and S9). Similarly, 21 ASVs from in situ adult sponges were shared with the + EM-FB samples, while 12 ASVs were shared with the -EM-FB samples (Fig. 5). These numbers were also found higher in + EM compared to -EM for t_0_, t_2_, t_3_, and t_4_. Additionally, a decreasing number of shared ASVs in + EM can be noticed over time (from 34 shared ASVs at t_1_, reaching 11 ASVs at t_6_, Fig. 5).

In addition to numbers of ASVs shared between the juveniles and the in situ adult sponges, the relative abundance of sequences from these ASVs and their taxonomy was investigated (Figure S10). At t_0_, the dominant shared ASVs within -EM gemmules belong to the *Terasakiellaceae* family (ASV6: unaffiliated genus, average relative abundance > 20%), while their relative abundance was lower in + EM samples (Figure S10). At t_1_ and t_2_, major shared ASVs (average relative abundance > 3%) identified belong to (i) the *Flavobacteriaceae* (ASV9: *Flavobacterium*), (ii) the *Rhodobacteraceae* (ASV18 and ASV21: *Defluviimonas* and *Tabrizicola*, respectively), and (iii) the *Terasakiellaceae* families (ASV6: unaffiliated genus). From t_2_ to t_4_, a higher relative percentage of shared ASVs from Gammaproteobacteria was observed, with the *Alteromonadaceae* (ASV14: *Rheinheimera*) and the *Comamonadaceae* (ASV65: *Paucibacter*). Finally, the *Rhodobacteraceae* (ASV6: *Flavobacterium*) were found mainly dominant in the t_5_ and t_6_ samples (Figure S10) When considering all shared ASVs together, important variations of their relative abundances were observed between and within the treatments, especially at t_1_, t_3_, and t_4_ within -EM + FB samples. However, despite these variations, a significant temporal decrease of percentages of shared ASVs was observed from t_0_ to t_6_ (ANOVA test: *p* < 0.001; Figure S10).

## Discussion

Our experiment was designed (i) to investigate the dynamics of bacterial communities during the first steps of the asexual cycle of *Spongilla lacustris*, and (ii) to decipher the transmission modes involved in the microbiome assembly. Only few studies experimentally investigated bacterial HT and VT hypotheses during the first steps of the sponge ontogeny [16, 54–56], and to date, our study brings the first insights for freshwater sponges. Through their sampling accessibility, but also the easiness of hatching and culturing, freshwater sponges such as *S. lacustris* or *Ephydatia muelleri*, are promising resources to better understand such mechanisms [28, 35]. Additionally, the gemmule surfaces sterilization protocol [34], provides a good experimental condition to test the VT scenario on the gemmule surface.

### Links between the ontogeny of *S. Lacustris* juveniles and the temporal dynamics of their bacterial communities

The development of *S. lacustris* juveniles after gemmule hatching observed during this experiment was similar to *E. muelleri* [34, 35] and *Ephydatia fluviatilis* [57, 58]. In these previous studies, five stages of juveniles development were described: (i) stage 1 (1–2 days after plating, pre- or just-hatching stage): the first stem cells are migrating out of the gemmules through the micropyle; (ii) stage 2 (1–3 days after hatching): the first tissues are growing around the gemmule husk, or on the substrate; (iii) stage 3 (2–4 days after hatching): canal system and choanocytes are formed; (iv) stage 4 (3–5 days after hatching): an osculum is starting to forms while the aquiferous system is still being organized; and (v) stage 5 (4–7 days after hatching): complex branched canals are formed and the osculum is developed. Based on this description and our observation, the first sampling time of our experiment (t_1_: 3 days after plating and 1 day after hatching) corresponds to stage 2, the second (t_2_: 5 days after hatching) corresponds to stage 4, while following ones (from t_3_ to t_6_: 9 days after hatching, and beyond) correspond to stage 5. Additionally, another important change in the development of the *S. lacustris* juveniles can be observed mainly after t_3_ with the colonization of *Chlorella*-like symbionts explaining the slight green coloration observed around the choanocyte chambers. The *Chlorella*-like colonization was found to be more important from t_4_ to t_6_, with a large proportion of tissues showing a green coloration more saturated. These results suggest that the symbiosis with the *Chlorella*-like symbionts is acquired before t_3_ but fully established in the whole juvenile body only after t_4_, under the light condition of our experiment (960 lx for day-light).

**Fig. 6:**
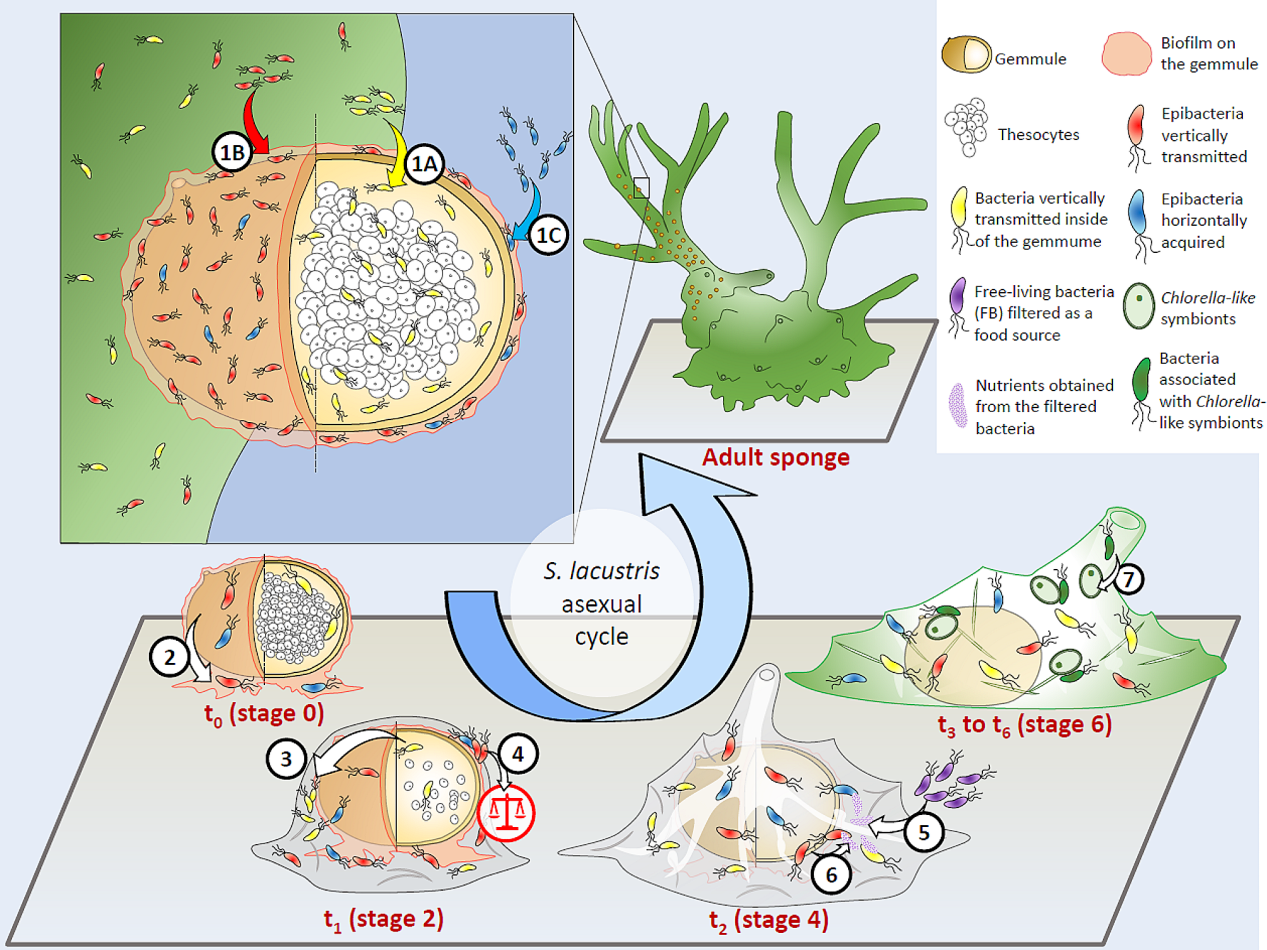
Illustration summarizing the potential transmission modes and the dynamics of the epibacterial community during the first life stages of the asexual cycle of *Spongilla lacustris*. The gemmule formed within the maternal tissue, encapsulate undifferentiated dormant cells: the thesocytes. Three transmission modes can be considered during the gemmule formation: (**1 A**) a vertical transmission of bacteria inside of the gemmule (e.g. *Terasakiellaceae* that might be associated with the thesocytes); (**1B**) a vertical transmission of bacteria on the gemmule surface within its biofilm (e.g. *Hydrogenophaga*), increasing the overall *α*-diversity; (**1 C**) a horizontal acquisition of ambient bacteria which are not directly associated with the maternal tissues (e.g. potential particle-attached planktonic colonizers). Stage 0: following its planktonic phase, the gemmule is in contact with a substrate, and (**2**) the epibacteria from the gemmule biofilm can colonize this new surface. These epibacteria (e.g. Bacteroidota such as *Flavobacterium*) might enhance the settlement of the gemmule and promote its hatching. Stage 1 to 2: the gemmule is hatching, with the thesocytes emerging out of the micropyle, together with the bacteria initially inside (**3**). The bacteria from the gemmule surface play an important role for the whole stability of the juvenile holobiont (**4**). Stage 3 to 4: the juvenile is developing its canal system and forming an osculum, while (**5**) the filtered free-living bacteria (FB) are used as a source of nutrients allowing an enrichment of copiotroph bacteria (**6**). Stage 5 and beyond: the increasing colonization of *Chlorella*-like symbionts, is associated with a shift of bacterial composition (**7**). Adult stage of *S. lacustris*: the tissues are forming a new generation of gemmules before winter. The different elements of the illustration are not to scale

When looking at the dynamics of the bacterial diversity after t_0_ and regardless of the treatment conditions, the main temporal changes can be observed in particular with a continuous shift from the *β*-diversity from t_1_ to t_6_. The specific diversity associated with t_1_ samples could be linked to the early development stage of these juveniles since the osculum is not formed yet, while the first cells are emerging around the gemmule husk, and colonizing the substrate (stage 2). This *β*-diversity difference seems to be explained in terms of composition by a higher relative abundance of Bacteroidota, and more specifically the *Flavobacteriaceae* (dominated by *Flavobacterium*) observed at t_1_ compared to the other following sampling times, but also compared to t_0_. These results suggest that a specific development of these pioneer bacterial taxa could be involved in these first steps following the hatching. Interestingly, diverse Bacteroidota strains (including one *Flavobacterium* sp.) were found to promote the settlement of the larvae of the marine sponge *Tedania* sp [55, 59]. These strains were either found to form biofilm enhancing the settlement of the larvae, or to excrete chemical cues inducing the larvae settlement through direct secretion or through the production of extracellular vesicles. A similar scenario (Fig. 6) could be considered for the settlement of the gemmules of *S. lacustris*, with an enrichment of *Flavobacterium* symbionts allowing the first sponge cells to better colonize their substrate (in our case: the gemmule husk and the flat bottom of the 24-well plate).

From t_2_ to t_4_ (stage 4 to 5), the formation of the osculum and the development of the aquiferous system are observed. The *β*-diversity differences with t_1_ can be linked to the ability of the sponge to actively filter the environment. Similar observations were demonstrated for the marine sponge *Crambe crambe* [56], as its prokaryotic community was found to change with regard to the osculum formation. In terms of composition, t_2_ to t_4_ samples are highlighted by a high abundance of Gammaproteobacteria, with three dominant families: *Comamonadaceae*, *Alteromonadaceae* and *Pseudomonadaceae*. These families are commonly occurring in freshwater sponges [21, 31, 60] and might be related to chemical defenses [61]. For example, *Comamonadaceae* were associated with defense mechanisms such as CRISPR and intracellular trafficking within *E. muelleri* [31], while diverse PKS genes were found within *Pseudomonadaceae* and *Alteromonadaceae* (dominant genus: *Rheinheimera*) associated with the freshwater sponges *Rezinkovia echinata* (Lake Baikal) and *E. fluviatilis* (Vinkeveense Plassen, the Netherlands), respectively [62, 63]. This specific bacterial community acquired during the formation of the aquiferous system and the first filter-feeding activity steps could be linked to the early development of bacterial symbionts acting for the chemical defense of the sponge.

From t_4_ to t_6_ (stage 5), the development of an important colonization of the *Chlorella*-like symbionts in the juvenile tissues, can be considered as a major factor explaining the differences with the previous sampling times (Fig. 6). Even if the bacterial communities associated with the *Chlorella*-like symbionts are similar to those of the freshwater sponge host [29], a part of this temporal change might be associated with the microalgal enrichment. Additionally, these microalgal symbionts provide a source of nutrients for the juveniles through the production of photosynthates which might also affect the bacterial composition in return.

### The bacterial community transmitted from the gemmule surface plays an important role in the microbiome stability of the juveniles

Our study tends to confirm the efficiency of the sterilization protocol of the gemmule surface developed by Leys et al. [34]. More precisely, no 16S rRNA sequences were successfully amplified when the gemmules were washed with hydrogen peroxide (-EM gemmules) and their DNA extracted without breaking the gemmule coating. Interestingly, the only way to amplify the 16S rRNA gene from the -EM gemmules was to break the gemmules coatings (by crushing them) before the DNA extraction. This result proves that gemmules host epibacteria within their core, and confirms that bacteria can be transmitted through the gemmule surface but also within the gemmule, as previously suggested [29, 31]. However, this result comes in opposition with culture-based observations from Rozenfeld and Curtis [64], suggesting a “spontaneous bacterial sterility” inside of *E. fluviatilis* gemmules. Here, we suggest that the methodology employed didn’t allow to correctly assess such sterility, considering the challenges associated with the culture of sponge-associated bacterial endosymbionts [65]. qPCR or microscopy analyses (e.g. FISH techniques or environmental SEM) could be considered in future studies to confirm our observation.

The sterilization of the gemmule surfaces, resulting in the absence of the EM, was found to be a major factor impacting the bacterial diversity of the gemmules and juveniles. More precisely, the removal of the EM reduces the *α*-diversity of the juveniles during the first steps of their growth, but also significantly changes the *β*-diversity all along the experiment (including before the hatching, at t_0_). Additionally, the *β*-diversity dispersion was significantly higher without the EM for the early stages. This result suggests that such an epibacterial community is not only distinct from the bacterial diversity transmitted inside of the gemmule, but also participates in the stability of the whole microbiome during early juvenile development (Fig. 6). The Anna Karenina principle adapted to sponge holobionts provides a good understanding on the importance of stable microbiomes [66]. This principle coined that high *β*-diversity dispersion could result from various dysbiosis scenarios induced by environmental stresses at the holobiont scale. The ability to regulate a stable and less dispersed microbiome over time is then an indicator of healthy microbiomes. In marine environments, the importance of such stability was demonstrated with the Haplosclerid *Petrosia ficiformis*, during the acquisition of host-specific cyanobacterial symbionts which might provide antioxidants protections for the host [67].

Differential analysis performed at t_0_ and t_1_ allowed to identify enriched taxa in + EM compared to -EM, to target bacterial taxa specifically associated with the gemmule surface. The *Comamonadaceae* and its main genus *Hydrogenophaga*, appear as major potential taxa attached to the gemmule surface (enriched in + EM condition). This genus is often described as autotrophic hydrogen oxidizers and denitrifiers [68], living in biofilms such as biofilm reactors [69–71] or epilithic biofilms in lake Baikal [72]. In this latter environment, this genus has also been observed within the freshwater sponge *Baikalospongia fungiformis* [73]. These observations suggest that such taxa might be adapted to colonize biofilms within freshwater sponges, such as the external surface of their gemmules. In this study, the *Comamonadaceae* family was already hypothesized to play an important role during the development of the aquiferous system. Consequently, the attachment of bacteria to the gemmule surface could constitute an important transmission mode shaping the development of juvenile holobionts.

Two hypotheses can be considered about the origin of taxa transmitted on the gemmule surface before the hatching: (i) a VT hypothesis where these taxa are vertically transmitted from the maternal tissue, or (ii) an “early HT” hypothesis where planktonic bacteria directly colonize the gemmule biofilm before or during the planktonic phase of the gemmule (Fig. 6). No stable association with the maternal cells would be involved in this case. This early HT hypothesis came then in contrast with both VT hypothesis and the traditional HT hypothesis, where in the latter the acquisition of planktonic colonizers occurs only once the juvenile has hatched, and filters the freshwater. A similar scenario to the early HT was suggested for *E. muelleri* [31], since epilithic biofilms were found to have similar bacterial communities to those from gemmules described in [35].

In our study, only a limited number of ASVs were found to be shared between the gemmules/juveniles and the in situ adult sponges, representing between 2 and 44.7% of the sequences. Surprisingly, almost no ASVs were shared between the gemmules/juveniles and the planktonic community (FF0.45 samples), suggesting that the remaining part of the community is acquired from (i) ultra-rare free-living taxa being undetected, or (ii) other sources than maternal tissues (VT) and free-living planktonic bacteria. In line with this hypothesis, we suggest that the colonization of the gemmule surface could also be achieved through an early HT involving planktonic particle-attached bacteria, even if their contribution could not be directly assessed through this study. As described in marine environments, the contribution of planktonic colonizers in the formation of biofilm can be challenging to assess, but particle-attached bacteria and the ultra-rare taxa are important to consider [74].

### The *Terasakiellaceae* as a dominant family vertically transmitted inside of the gemmules

The *β-*diversity analysis conducted at t_0_ indicated a significant effect of the sterilization of the surface, confirming the difference of bacterial composition transmitted inside of the gemmules compared to their surface. The *Terasakiellaceae* family are good candidates for these types of taxa transmitted specifically from the maternal tissues to the inside of a gemmule. More precisely, the differential analysis revealed their specificity for the -EM samples at t_0_ and t_1_. For the same sampling times, this family also gathers the dominant ASVs shared between the adult sponge and the -EM gemmules. Additionally, within the in situ adult sponge samples, this family was found mainly dominant in the M2 samples but less abundant in the M3 samples. This observation can be explained since the formation of the gemmules and their thesocytes mainly occurred in late November (after M2), while in December (M3) most of the tissue from the adult sponge was found absent and full of gemmules within its skeleton. As the in situ adult sponge samples were extracted mostly with unbroken gemmules, the formation of the gemmules could lead to a lower relative abundance of *Terasakiellaceae* in the M3 sponge tissues, which are mostly transmitted and located within the gemmules. Consequently, these specific taxa might be important endosymbionts, transmitted to the thesocytes within the gemmule (Fig. 6). Further studies are needed to confirm their location and their role as potential endosymbionts.

*Terasakiellaceae* were found in the primmorphs of the diseased freshwater sponge *Lubomirskia baikalensis* [75], but also within marine holobionts such as the sponge *Suberites massa* [76], as well as an important diversity of corals [77–80]. Taxa within this family are also known as nitrogen fixers [81], and may play an important role for the nitrogen regulation within the gemmule. Others taxa enriched in the -EM samples such as the *Rhizobiaceae* family and its dominant genus *Rhizobium* (covering also *Allorhizobium*, *Neorhizobium* and *Parararhizobium*) known for its denitrification role [82, 83], might also play similar functions within the gemmule. To date, little is known about the role of bacterial symbionts within sponge cells undergoing diapause states such as the thesocytes cells within the gemmules. The expression of glutamine metabolism, apoptotic process, and oxidation-reduction system was found to be specific to stage 0 [35]. Further studies are needed to better investigate the potential link between these specific metabolisms and these putative endosymbionts stored in the gemmules.

As the experiment were performed with gemmules collected from the same specimen (one single gemmule patch collected), the understanding of the effect of intra-specific variations in the symbiont acquisition is limited in this study. Further experiments are needed with biological replicates of maternal individuals, leading to different lineage groups of juveniles.

### Effect of the presence of exogenous free-living bacteria during the juveniles growth: horizontal acquisition of symbionts, or additional food source (FS)?

Another major result of this study was the effect of the absence/presence of ambient free-living bacteria on the bacterial *β*-diversity of the juveniles. These differences can be explained through two hypotheses: (i) an HT hypothesis, related to the acquisition of bacterial symbionts from the medium, and (ii) a food source (FS) hypothesis, where the bacterioplankton is filtered by the sponge for its nutrition. In this scenario, this specific food source would be linked to a different physiology of the juveniles impacting indirectly their microbiome. As mainly discussed for marine sponges, the delineation between these two processes can be hard to distinguish [7].

In this study, only three low abundant ASVs (*Polynucleobacter*, Candidatus *Limnoluna*, and *Bradyrhizobium*; each < 0.04%) were occasionally shared between the prefiltered water (FF0.45 samples) and the juvenile sponges, suggesting that the horizontal acquisition of FL bacteria by the juveniles was nearly nonexistent during this experiment. The effect of the presence of the FL bacteria in the medium on the *β*-diversity is then more likely explained by the FS hypothesis (Fig. 6). Additionally, the *α*-diversity analysis also goes in favor of the rejection of the HT hypothesis, since the richness (estimated with Chao1 index) of the + FB juveniles was not significantly higher compared to the -FB.

From t_1_ to t_3_, the difference of *β*-diversity between + FB and -FB samples was mainly observed under the -EM condition, while being less important in + EM. Consequently, the absence of epibiotic microbiome (EM) increases the effect of the absence of ambient free-living bacteria (FB). Their absence in the medium might then represent cumulative stress to the juveniles growing with an unstable microbiome due to the EM absence. In line with the FS hypothesis, the absence of FB as a food source might explain this as an additional source of stress in + EM, leading to a distinct bacterial community.

As the differences between + FB and -FB were linked to the food source availability, these differences can be investigated in terms of trophic strategy within the bacterial community. Indeed, the juveniles growing with more food could provide more nutrients for their associated microbiome. Interestingly, this assumption appears to be particularly consistent with the specific taxa found enriched in + FB or -FB samples, based on differential analyses at t_1_. For example, several + FB enriched taxa such as *Oxalobacteraceae* (within + EM samples), but also *Pseudomonas* and *Flavobacterium* (within -EM samples) are classically known as copiotroph [84, 85]. Conversely, -FB enriched taxa such as *Rheinemera* (in + EM) or *Brevundimonas* and *Caulobacter* (in both -EM and + EM) are typically oligotrophic bacteria [85, 86]. This observation strongly supports the FS hypothesis, indicating that in the absence of FB, less nutrients can be provided to the microbiome, resulting in a higher relative abundance of oligotrophic taxa. Conversely, the presence of FB turned into a source of organic matter by the juveniles, could favor copiotrophs that could take advantage of this condition and quickly grow within the sponge (i.e. r-strategists bacteria).

Finally, even if almost no ASVs from the prefiltered water were found to be horizontally acquired within the juveniles, a slightly higher number of these free-living ASVs were found to be shared with the *in-situ* sponges (17 in total, with dominant ones belonging to *Sporichthyaceae*). This observation suggests that horizontal acquisition within *S. lacustris* could still occasionally happen after a longer term of development. Further studies on these transmissions in natural conditions could provide relevant insight to better consider the complete dynamics of the holobiont through its full life cycle.

## Conclusion

The bacterial diversity within *S. lacustris* juveniles was found to be shaped by three factors: the life cycle stage, the presence of epibacteria on the gemmule, and finally the presence of ambient free-living bacteria. The osculum formation together with the development of a canal system, could lead to an active filtration of the environment which might induce a change in the microbiome *β*-diversity. Thereafter, the colonization of the *Chlorella*-like symbionts could also provide an additional niche for the development of new bacterial symbionts within the holobiont.

Our study revealed a complex diversity of microbial acquisition modes within the *S. lacustris* holobiont model. For instance, multiple vertical acquisition scenarios can be considered with both transmission within the gemmule or on its surface. Importantly, the transmission of the microbiome on the gemmule surface was found to be essential for the whole holobiont stability during the first days. In line with the Anna Karenina principle [66], the absence of these epibacterial communities might represent a stress condition. While a recruitment of free-living bacteria by filtration of the juveniles was found to be nearly impossible, an alternative horizontal transmission scenario can however be considered with a colonization of planktonic bacteria on the gemmule biofilm, before the hatching. The community of the gemmule biofilm would then be composed of both vertically and horizontally transmitted bacteria, in line with the mixed-mode transmission hypothesis [9]. Such diversity and complexity of transmission modes need to be better considered in future studies, and our results associated with the importance of the microbiome on the gemmule surface provide new perspectives that could also be investigated for sponge larvae.

### Electronic supplementary material

Below is the link to the electronic supplementary material.

Supplementary Material 1

## Data Availability

16S rRNA gene sequences were deposited and are publicly available in the NCBI Sequences Read Archive (SRA) under the BioProject ID PRJNA1077127, accession number. The R scripts used for all the 16S rRNA gene metabarcoding analyses can be found at https://github.com/BenoitPAIX/Gemmules_microbiome.

## Abbreviations

ASV: Amplicon Sequence Variant
EM: Epibiotic Microbiome
FF0.2: Freshwater Filtered upon 0.2 μm pore size filters
FF0.45: Freshwater Filtered upon 0.45 μm pore size filters
FB: Free-living Bacteria
FS: Food Source
HT: Horizontal Transmission
SD: Standard Deviation
VT: Vertical Transmission
