# Supplementary material for: Dynamics, diversity, and roles of bacterial transmission modes during the first asexual life stages of the freshwater sponge Spongilla lacustris
Source: Environ Microbiome. 2024 Jun 8;19:37. doi: 10.1186/s40793-024-00580-7 (PMC11162577; doi:10.1186/s40793-024-00580-7)

**Supplementary informations**

**Dynamics, diversity, and roles of bacterial transmission modes during the first asexual life stages of the freshwater sponge *Spongilla lacustris***

Benoit Paix^1,2*^, Elodie van der Valk^1,3^, Nicole J. de Voogd^1,3*^

^1^ Naturalis Biodiversity Center, Leiden, The Netherlands

^2^ Present address: UMR CARRTEL, INRAE - Université Savoie Mont-Blanc, Thonon-les-Bains, France

^3^ Institute of Biology (IBL), Leiden University, The Netherlands

* Corresponding authors: [benoit.paix@gmail.com](mailto:benoit.paix@gmail.com), [n.j.de.voogd@biology.leidenuniv.nl](https://webmail.campus.leidenuniv.nl/owa/redir.aspx?C=Za4ICcaZ4IKSUxkpdSSDv54aIPa-tU-W6kd9Q4htsAtTTZjeU0nWCA..&URL=mailto%3an.a.soudzilovskaia%40cml.leidenuniv.nl)

#

# Supplementary material and methods notes

## Supplementary note S1. Detailed description of the experiment’s timeline

| **Day** | **Sampling** | **After sampling** |
| --- | --- | --- |
| Day 0 | t_0_ samples (unplated gemmules) | Plating of the gemmules for t_1_ to t_6_ |
| Day 3 | t_1_ samples | Refreshing with FF0.45 and FF0.2 |
| Day 7 | t_2_ samples | Refreshing with FF0.45 and FF0.2  Sampling of free-living cells from the FF0.45 filtrate with 0.2μm pore size filters |
| Day 10 | - | Refreshing with FF0.45 and FF0.2 |
| Day 11 | t_3_ samples | - |
| Day 14 | - | Refreshing with FF0.45 and FF0.2  Sampling of free-living cells from the FF0.45 filtrate with 0.2μm pore size filters |
| Day 17 | t_4_ samples | Refreshing with FF0.45 and FF0.2  Sampling of free-living cells from the FF0.45 filtrate with 0.2μm pore size filters |
| Day 21 | - | Refreshing with FF0.45 and FF0.2  Sampling of free-living cells from the FF0.45 filtrate with 0.2μm pore size filters |
| Day 24 | t_5_ samples | Refreshing with FF0.45 and FF0.2 |
| Day 28 | - | Refreshing with FF0.45 and FF0.2  Sampling of free-living cells from the FF0.45 filtrate with 0.2μm pore size filters |
| Day 31 | t_6_ samples | - |

## Supplementary note S2. Tests of DNA extractions and PCR amplifications on gemmule samples

The gemmule coating was found to be resistant to the mechanical breaking method (beat beating) used for the FastDNA™ SPIN Kit for Soil (40 seconds in the FastPrep instrument at a speed setting of 6.0 m/s). Consequently, the effect of breaking the gemmules coating (by crushing them using the top of 10μL filter pipette tips) was tested. We hypothesized that DNA extraction performed without breaking the coating could not allow the detection of bacterial DNA inside the gemmule.

To assess the efficiency of the sterilization of gemmules suggested by Leys et al., 2019 through the washing with H_2_O_2_ as described, we performed PCR targeting the 16S rRNA gene (same primers and PCR conditions as in the main study) of three types of gemmules: (i) gemmules washed with H_2_O_2_ and broken before DNA extraction (named “+H_2_O_2__broken”, n=4), (ii) gemmules washed with H_2_O_2_ and let intact before the DNA extraction (named “+H_2_O_2__unbroken”, n=4), (iii) gemmules washed without H2O2 and let intact before the DNA extraction (named “-H_2_O_2__unbroken”, n=4).

The gel electrophoresis results indicated the presence of 16S rRNA gene amplicons for the +H_2_O_2__broken and -H_2_O_2__unbroken gemmules, while no amplicons were detected for the +H_2_O_2__unbroken. These gel electrophoresis results were confirmed using the Tapestation 4150 (Kit HSD 5000, Agilent Technologies, Santa Clara, CA, United States). The absence of amplicons in +H_2_O_2__unbroken samples suggests that the gemmule surface is sterilized and the bacterial DNA from the inside of the gemmule is not extracted. Consequently, this result confirms the efficiency of the sterilization. When the gemmules are broken, the 16S rRNA gene amplicons are always detected, suggesting that the inside of the gemmules are always hosting bacterial symbionts.

Supplementary analyses combining microscopy imaging and qPCR experiments can be considered in the future to confirm these results.

# Supplementary tables

## Table S1. Summary of the sample list.

The shape and color codes correspond to those used in the experimental workflow (**Figure 1**), boxplots, and NMDS. NA: Non-applicable.

| **Sample type and shape code** | | **Sampling time and color code** | | **Treatment** | | | **Replicates numbers** |
| --- | --- | --- | --- | --- | --- | --- | --- |
|  |  |  |  | **± EM** | **± FB** | **Color code** |  |
| Filtered freshwater | ▽ | t2 |  | NA | NA | NA | 1 |
|  |  | t3 |  |  |  |  | 1 |
|  |  | t4 |  |  |  |  | 1 |
|  |  | t5 |  |  |  |  | 1 |
| *In situ* adult sponge | ◇ | M1 |  | NA | NA | NA | 4 |
|  |  | M2 |  |  |  |  | 4 |
|  |  | M3 |  |  |  |  | 4 |
| Gemmule | □ | t0 |  | - EM | NA |  | 4 |
|  |  |  |  | +EM |  |  | 5 |
| *In vitro* juvenile sponge | ○ | t1 |  | - EM | -FB |  | 5 |
|  |  |  |  |  | +FB |  | 4 |
|  |  |  |  | +EM | -FB |  | 4 |
|  |  |  |  |  | +FB |  | 5 |
|  |  | t2 |  | - EM | -FB |  | 5 |
|  |  |  |  |  | +FB |  | 4 |
|  |  |  |  | +EM | -FB |  | 5 |
|  |  |  |  |  | +FB |  | 5 |
|  |  | t3 |  | - EM | -FB |  | 5 |
|  |  |  |  |  | +FB |  | 4 |
|  |  |  |  | +EM | -FB |  | 5 |
|  |  |  |  |  | +FB |  | 5 |
|  |  | t4 |  | - EM | -FB |  | 5 |
|  |  |  |  |  | +FB |  | 5 |
|  |  |  |  | +EM | -FB |  | 5 |
|  |  |  |  |  | +FB |  | 4 |
|  |  | t5 |  | - EM | -FB |  | 5 |
|  |  |  |  |  | +FB |  | 4 |
|  |  |  |  | +EM | -FB |  | 4 |
|  |  |  |  |  | +FB |  | 3 |
|  |  | t6 |  | - EM | -FB |  | 4 |
|  |  |  |  |  | +FB |  | 5 |
|  |  |  |  | +EM | -FB |  | 4 |
|  |  |  |  |  | +FB |  | 5 |

## Table S2. Results of the Kruskal-Wallis test conducted with the α-diversity indexes, with the sample types as factors of comparison.

|  | χ^2^ | Degree of freedom | *p*-value |
| --- | --- | --- | --- |
| Chao1 | 7.424057 | 3 | **0.059542** |
| Shannon | 4.700287 | 3 | **0.195106** |
| Pielou | 14.7937 | 3 | **0.002002** |

## Table S3. Results of the Kruskal-Wallis test conducted with the α-diversity measures of juvenile sponge samples, with the sampling time combined with ±EM as a comparison factor.

|  | χ^2^ | Degree of freedom | *p*-value |
| --- | --- | --- | --- |
| Chao1 | 64.22240002 | 13 | **9.12E-09** |
| Shannon | 74.05576807 | 13 | **1.43E-10** |
| Pielou | 70.6140741 | 13 | **6.19E-10** |

##

## Table S4. Results of the multivariate statistical analyses performed with all samples

**A.** Results of the PERMANOVA test conducted with the sample type factor. D. f; F; R^2^ and *p* correspond to degrees of freedom; F ratio; coefficient of determination and *p*-value, respectively.

|  | **D.f.** | **Sum of squares** | **R^2^** | **F** | ***p*** |
| --- | --- | --- | --- | --- | --- |
| **Sample type** | 3 | 9.405459 | 0.195338 | 10.51949 | **0.001** |
| **Residual** | 130 | 38.74426 | 0.804662 | NA | NA |
| **Total** | 133 | 48.14972 | 1 | NA | NA |

**B.** Summary of the *p*-values obtained from the multivariate pairwise test conducted with the sample type factor.

|  | ***In vitro* juvenile sponge** | ***In situ* adult sponge** | **Filtered freshwater** |
| --- | --- | --- | --- |
| **Gemmule** | **0.001** | **0.001** | **0.004** |
| ***In vitro* juvenile sponge** | - | **0.004** | **0.001** |
| ***In situ* adult sponge** | - | - | **0.001** |

##

## Table S5. Results of the multivariate statistical analyses performed on juvenile sponge samples

**A**. Result of the 3-way PERMANOVA test conducted with the sampling time, ±EM, and ±FB as factors. D. f; F; R^2^ and *p* correspond to degrees of freedom; F ratio; coefficient of determination and *p*-value, respectively

|  | **D.f.** | **Sum of squares** | **R^2^** | **F** | ***p*** |
| --- | --- | --- | --- | --- | --- |
| Sampling time | 5 | 6.972582 | 0.198154 | 6.941356 | **0.001** |
| ± EM | 1 | 3.254917 | 0.092501 | 16.2017 | **0.001** |
| ± FB | 1 | 0.903746 | 0.025684 | 4.498491 | **0.001** |
| Sampling time * ± EM | 5 | 2.798326 | 0.079526 | 2.785794 | **0.001** |
| Sampling time * ± FB | 5 | 1.732174 | 0.049227 | 1.724417 | **0.001** |
| ± EM * ± FB | 1 | 0.770522 | 0.021897 | 3.835358 | **0.001** |
| Sampling time * ± EM * ± FB | 5 | 1.678989 | 0.047715 | 1.67147 | **0.001** |
| Residual | 85 | 17.07648 | 0.485296 | NA | NA |
| Total | 108 | 35.18773 | 1 | NA | NA |

**B.** Summary of the *p*-values obtained from the multivariate pairwise comparison between sampling times

|  | **t2** | **t3** | **t4** | **t5** | **t6** |
| --- | --- | --- | --- | --- | --- |
| **t1** | **0.002** | **0.001** | **0.001** | **0.001** | **0.001** |
| **t2** | - | **0.261** | **0.005** | **0.001** | **0.001** |
| **t3** | - | - | **0.215** | **0.001** | **0.001** |
| **t4** | - | - | - | **0.002** | **0.001** |
| **t5** | - | - | - | - | **0.075** |

**C**. Summary of the *p*-values obtained from the multivariate pairwise comparison between treatments

|  | **-EM-FB** | **+EM+FB** | **+EM-FB** |
| --- | --- | --- | --- |
| **-EM+FB** | **0.006** | **0.001** | **0.001** |
| **-EM-FB** | - | **0.001** | **0.001** |
| **+EM+FB** | - | - | **0.154** |

## Table S6. Results of the multivariate statistical analyses conducted with the microbiome datasets of juvenile sponge samples, at each sampling time independently

**A**. Result of the 1-way PERMANOVA test (t_0_) and 2-way PERANOVA tests (t_1_ to t_6_), conducted at each sampling time with ±EM and ±FB as factors. D. f; F; R^2^ and *p* correspond to degrees of freedom; F ratio; coefficient of determination and *p*-value, respectively

|  | | **D.f.** | **Sum of squares** | **R^2^** | **F** | ***p*** |
| --- | --- | --- | --- | --- | --- | --- |
| t_0_ | ±EM | 1 | 0.440197 | 0.314873 | 3.217085 | **0.006** |
|  | Residual | 7 | 0.957816 | 0.685127 | NA | NA |
|  | Total | 8 | 1.398013 | 1 | NA | NA |
| t_1_ | ±EM | 1 | 0.968039 | 0.201204 | 4.649238 | **0.001** |
|  | ±FB | 1 | 0.459535 | 0.095513 | 2.207025 | **0.01** |
|  | ±EM * :±FB | 1 | 0.468662 | 0.09741 | 2.25086 | **0.006** |
|  | Residual | 14 | 2.915005 | 0.605874 | NA | NA |
|  | Total | 17 | 4.811241 | 1 | NA | NA |
| t_2_ | ±EM | 1 | 1.184402 | 0.280003 | 8.027761 | **0.001** |
|  | ±FB | 1 | 0.360457 | 0.085215 | 2.44314 | **0.02** |
|  | ±EM * ±FB | 1 | 0.472029 | 0.111592 | 3.199365 | **0.005** |
|  | Residual | 15 | 2.213075 | 0.52319 | NA | NA |
|  | Total | 18 | 4.229963 | 1 | NA | NA |
| t_3_ | ±EM | 1 | 1.380901 | 0.292065 | 8.576907 | **0.001** |
|  | ±FB | 1 | 0.540712 | 0.114362 | 3.358414 | **0.005** |
|  | ±EM * ±FB | 1 | 0.391418 | 0.082786 | 2.431136 | **0.015** |
|  | Residual | 15 | 2.415034 | 0.510787 | NA | NA |
|  | Total | 18 | 4.728066 | 1 | NA | NA |
| t_4_ | ±EM | 1 | 0.68379 | 0.13206 | 2.855086 | **0.003** |
|  | ±FB | 1 | 0.42038 | 0.081188 | 1.75525 | **0.032** |
|  | ±EM * ±FB | 1 | 0.481212 | 0.092936 | 2.009246 | **0.021** |
|  | Residual | 15 | 3.592482 | 0.693815 | NA | NA |
|  | Total | 18 | 5.177864 | 1 | NA | NA |
| t_5_ | ±EM | 1 | 0.812263 | 0.200413 | 4.004134 | **0.001** |
|  | ±FB | 1 | 0.442079 | 0.109076 | 2.179273 | **0.008** |
|  | ±EM * ±FB | 1 | 0.364323 | 0.089891 | 1.795969 | **0.03** |
|  | Residual | 12 | 2.434273 | 0.600619 | NA | NA |
|  | Total | 15 | 4.052938 | 1 | NA | NA |
| t_6_ | ±EM | 1 | 1.025031 | 0.196551 | 4.092398 | **0.001** |
|  | ±FB | 1 | 0.411573 | 0.07892 | 1.643189 | **0.022** |
|  | ±EM * ±FB | 1 | 0.271867 | 0.052131 | 1.085419 | **0.267** |
|  | Residual | 14 | 3.506607 | 0.672398 | NA | NA |
|  | Total | 17 | 5.215077 | 1 | NA | NA |

**B.** Summary of the *p*-values obtained from the multivariate pairwise comparison between treatments, for each sampling time.

|  | | -EM | | |
| --- | --- | --- | --- | --- |
| t_0_ | +EM | **0.01** | | |
|  | | | | |
|  | | +EM-FB | -EM+FB | -EM-FB |
| t_1_ | +EM+FB | **0.009** | **0.008** | **0.007** |
|  | +EM-FB | - | **0.024** | **0.008** |
|  | -EM+FB | - | - | **0.011** |
| t_2_ | +EM+FB | **0.01** | **0.011** | **0.015** |
|  | +EM-FB | - | **0.006** | **0.008** |
|  | -EM+FB | - | - | **0.107** |
| t_3_ | +EM+FB | **0.01** | **0.008** | **0.009** |
|  | +EM-FB | - | **0.009** | **0.01** |
|  | -EM+FB | - | - | **0.01** |
| t_4_ | +EM+FB | **0.201** | **0.118** | **0.036** |
|  | +EM-FB | - | **0.025** | **0.009** |
|  | -EM+FB | - | - | **0.013** |
| t_5_ | +EM+FB | **0.036** | **0.035** | **0.01** |
|  | +EM-FB | - | **0.031** | **0.013** |
|  | -EM+FB | - | - | **0.01** |
| t_6_ | +EM+FB | **0.09** | **0.005** | **0.009** |
|  | +EM-FB | - | **0.014** | **0.032** |
|  | -EM+FB | - | - | **0.133** |

## Table S7. Results of the ANOVA tests conducted on the *β*-diversity dispersion (distance to centroids) of gemmules and juvenile sponges, at each sampling time separately, using ±EM and the ±FB as comparison factors

D.f.; F; and *p* correspond to degrees of freedom; F ratio; and *p*-value, respectively

|  | | **D.f.** | **Sum of squares** | **Mean square** | **F** | ***p*** |
| --- | --- | --- | --- | --- | --- | --- |
| t_0_ | ±EM | 1 | 0.00282 | 0.00282 | 2.442062 | **0.162094** |
|  | Residuals | 7 | 0.008084 | 0.001155 | NA | NA |
| t_1_ | **±EM** | 1 | 0.151627 | 0.151627 | 39.94246 | **1.89E-05** |
|  | ±FB | 1 | 0.000203 | 0.000203 | 0.053424 | **0.820553** |
|  | ±EM * ±FB | 1 | 0.010826 | 0.010826 | 2.851971 | **0.113401** |
|  | Residuals | 14 | 0.053146 | 0.003796 | NA | NA |
| t_2_ | **±EM** | 1 | 0.038847 | 0.038847 | 10.88796 | **0.004862** |
|  | ±FB | 1 | 0.000756 | 0.000756 | 0.211881 | **0.651899** |
|  | ±EM * ±FB | 1 | 0.004796 | 0.004796 | 1.34433 | **0.264396** |
|  | Residuals | 15 | 0.053519 | 0.003568 | NA | NA |
| t_3_ | ±EM | 1 | 0.010596 | 0.010596 | 4.2788 | **0.056284** |
|  | ±FB | 1 | 0.000885 | 0.000885 | 0.357301 | **0.558926** |
|  | ±EM * ±FB | 1 | 1.55E-05 | 1.55E-05 | 0.006254 | **0.938011** |
|  | Residuals | 15 | 0.037145 | 0.002476 | NA | NA |
| t_4_ | ±EM | 1 | 0.021569 | 0.021569 | 1.922443 | **0.185851** |
|  | ±FB | 1 | 0.014654 | 0.014654 | 1.306172 | **0.271003** |
|  | ±EM * ±FB | 1 | 0.004449 | 0.004449 | 0.396522 | **0.538365** |
|  | Residuals | 15 | 0.16829 | 0.011219 | NA | NA |
| t_5_ | ±EM | 1 | 0.001252 | 0.001252 | 1.02256 | **0.331868** |
|  | **±FB** | 1 | 0.015518 | 0.015518 | 12.67439 | **0.003923** |
|  | ±EM * ±FB | 1 | 0.00403 | 0.00403 | 3.291773 | **0.094691** |
|  | Residuals | 12 | 0.014692 | 0.001224 | NA | NA |
| t_6_ | ±EM | 1 | 0.000115 | 0.000115 | 0.023873 | **0.879413** |
|  | ±FB | 1 | 0.005098 | 0.005098 | 1.060325 | **0.320604** |
|  | ±EM * ±FB | 1 | 0.001109 | 0.001109 | 0.230688 | **0.638433** |
|  | Residuals | 14 | 0.067315 | 0.004808 | NA | NA |

#

# Supplementary figures

## Figure S1. Pictures of the juveniles at the different sampling times. Red arrows indicate the osculum


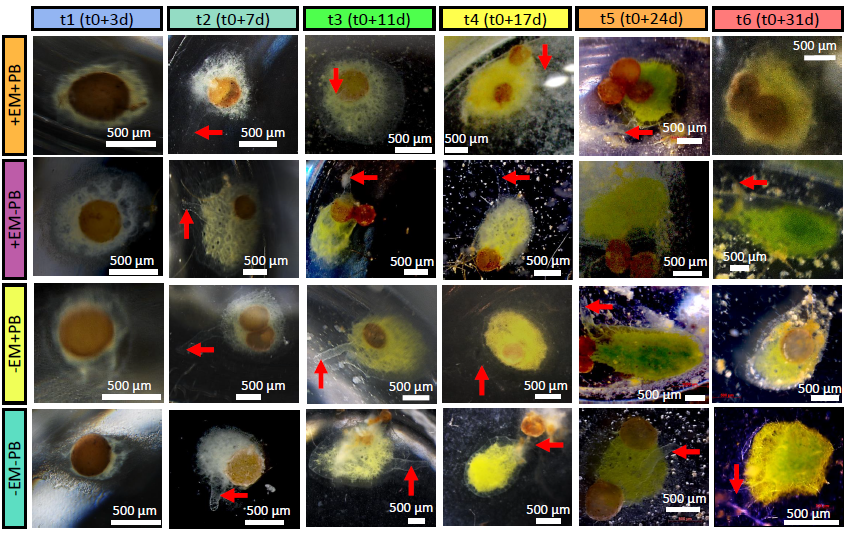


## Figure S2. *α*-diversity measures (A: Chao1 index, B: Shannon index, C: Pielou index) of the bacterial communities associated with the different sample types (*in situ* adult sponge, gemmules, *in vitro* juveniles, and filtered freshwater [FF0.45]).

*p*-values corresponded to the results of Kruskal-Wallis tests using the sample type as a comparison factor. Lowercase indices (a and b) represent results from the Wilcoxon tests comparing each sample type.


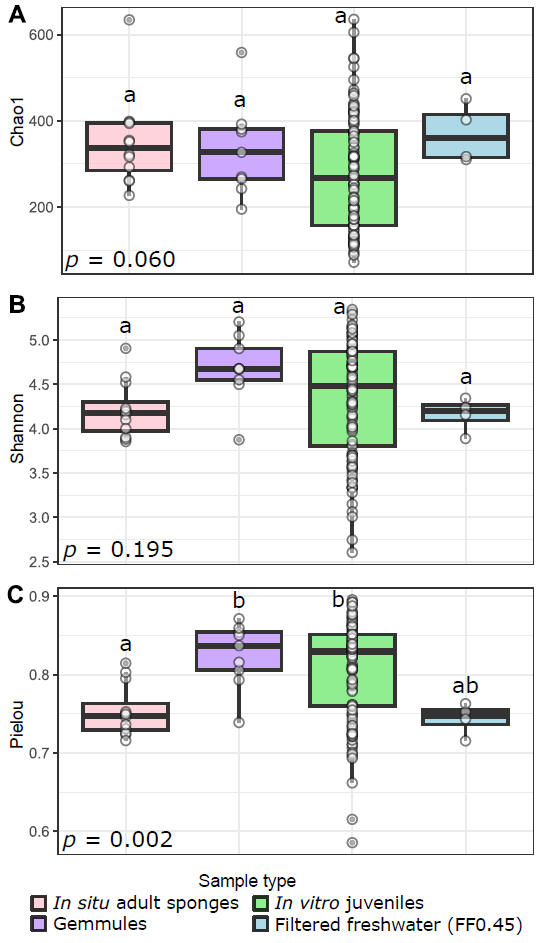


## Figure S3. Dynamics of the α-diversity measures (A: Chao1 index and B: Pielou index) of the bacterial communities associated with the gemmules (t_0_) and the *in vitro* juvenile sponges (t_1_ to t_6_).

Lowercase indices (a to d) represent results from the Wilcoxon test comparing ±EM samples within each time.


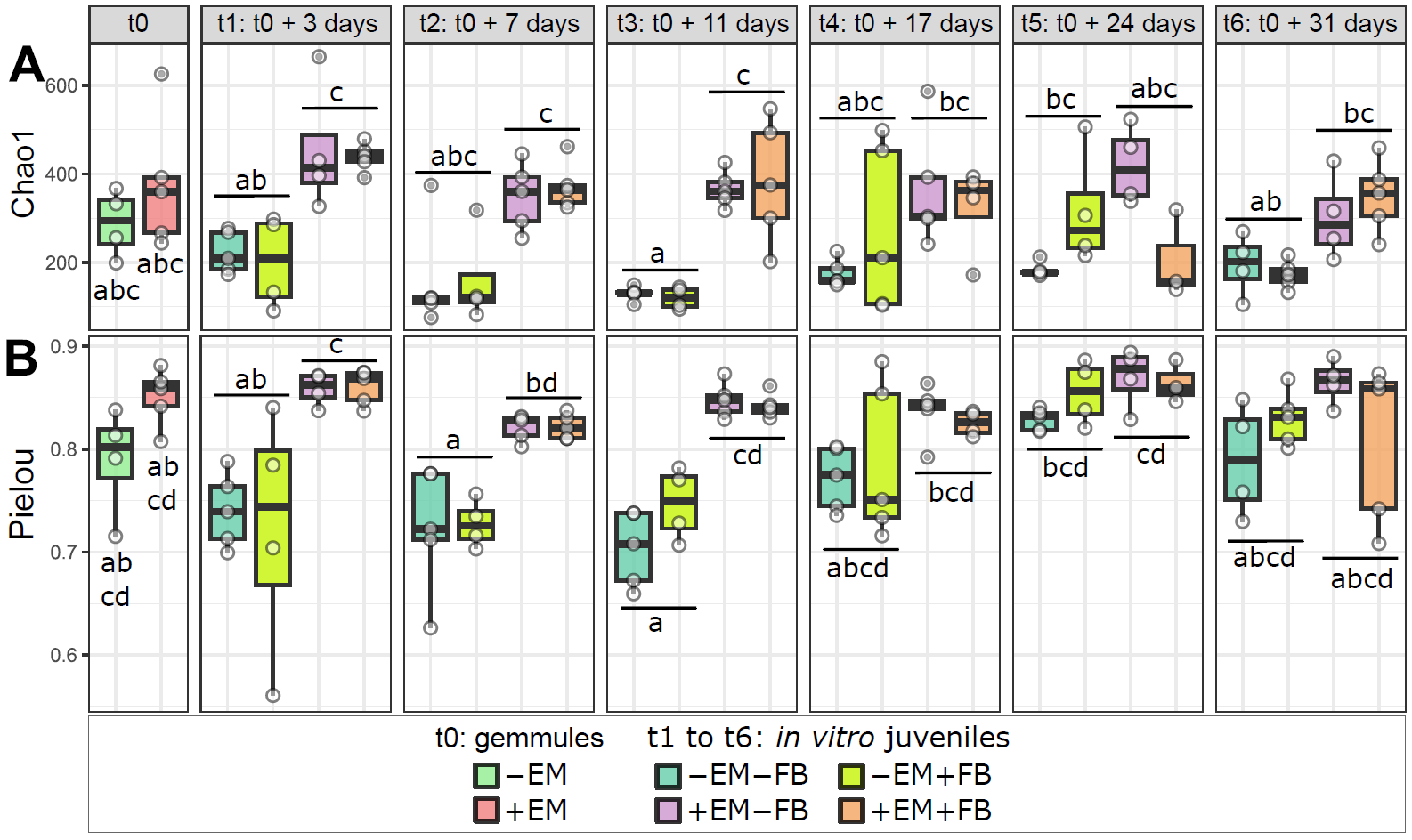


## Figure S4. *β*-diversity of bacterial communities (Bray-Curtis dissimilarity).

The NMDS was plotted with all samples represented according to their sample type (shape) and the treatments for the gemmules and juveniles (color).


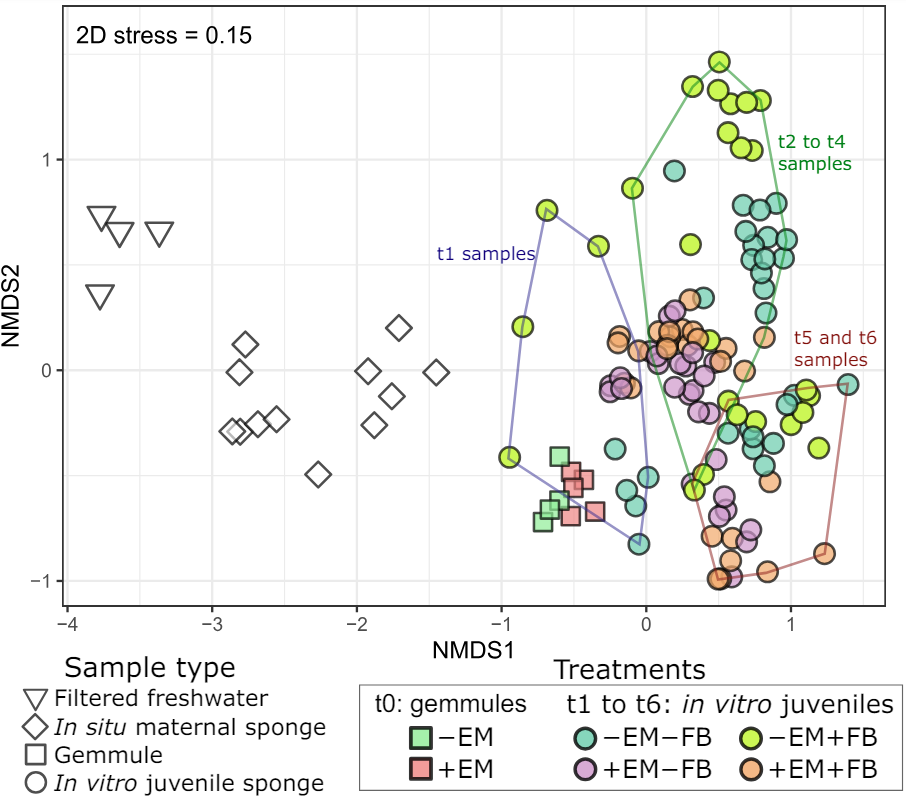


## Figure S5. Dispersion dynamics of the *β*-diversity of gemmules (t_0_) and *in vitro* juvenile sponges (t_1_ to t_6_) associated bacterial communities.

The *β***-**dispersion (beta-disper) was calculated using the distance to centroid between the Bray-Curtis dissimilarity of replicates from each treatment at each sampling time. *p*-values corresponded to the results of one-way ANOVA (t_0_) and two-way ANOVA (t_1_ to t_6_) using the sampling ±EM and ±FB as comparison factors. Lowercase indices (a and b) represent results from the HSD Tukey’s tests comparing each treatment within each time.


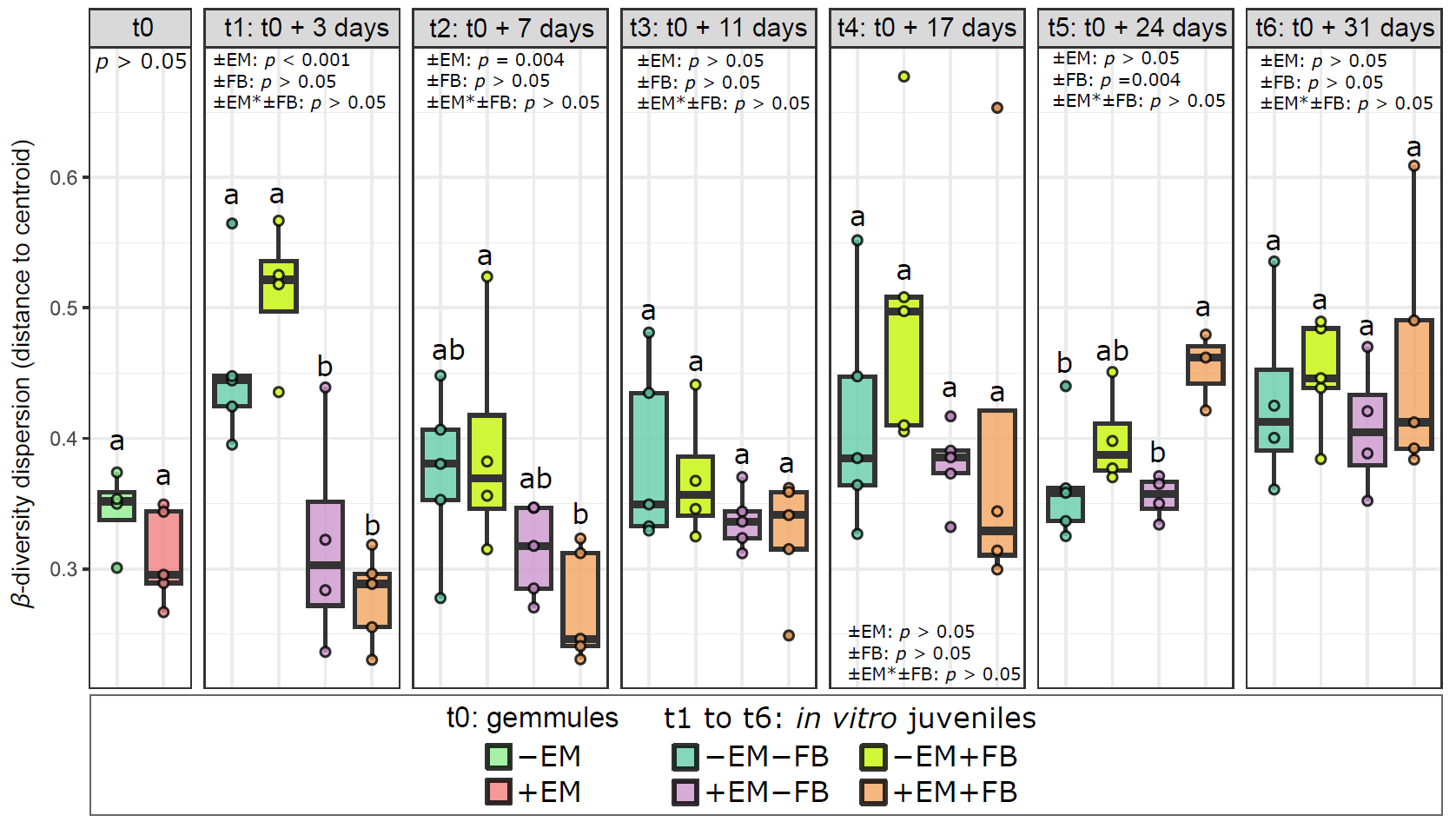


## Figure S6. Bacterial community composition at the family level.

**A**. Barplots of the relative percentages of the bacterial community of filtered freshwater, *in situ* adult sponges, and gemmule samples. **B**. Barplots of the relative percentages of the bacterial community of the *in vitro* juvenile samples.


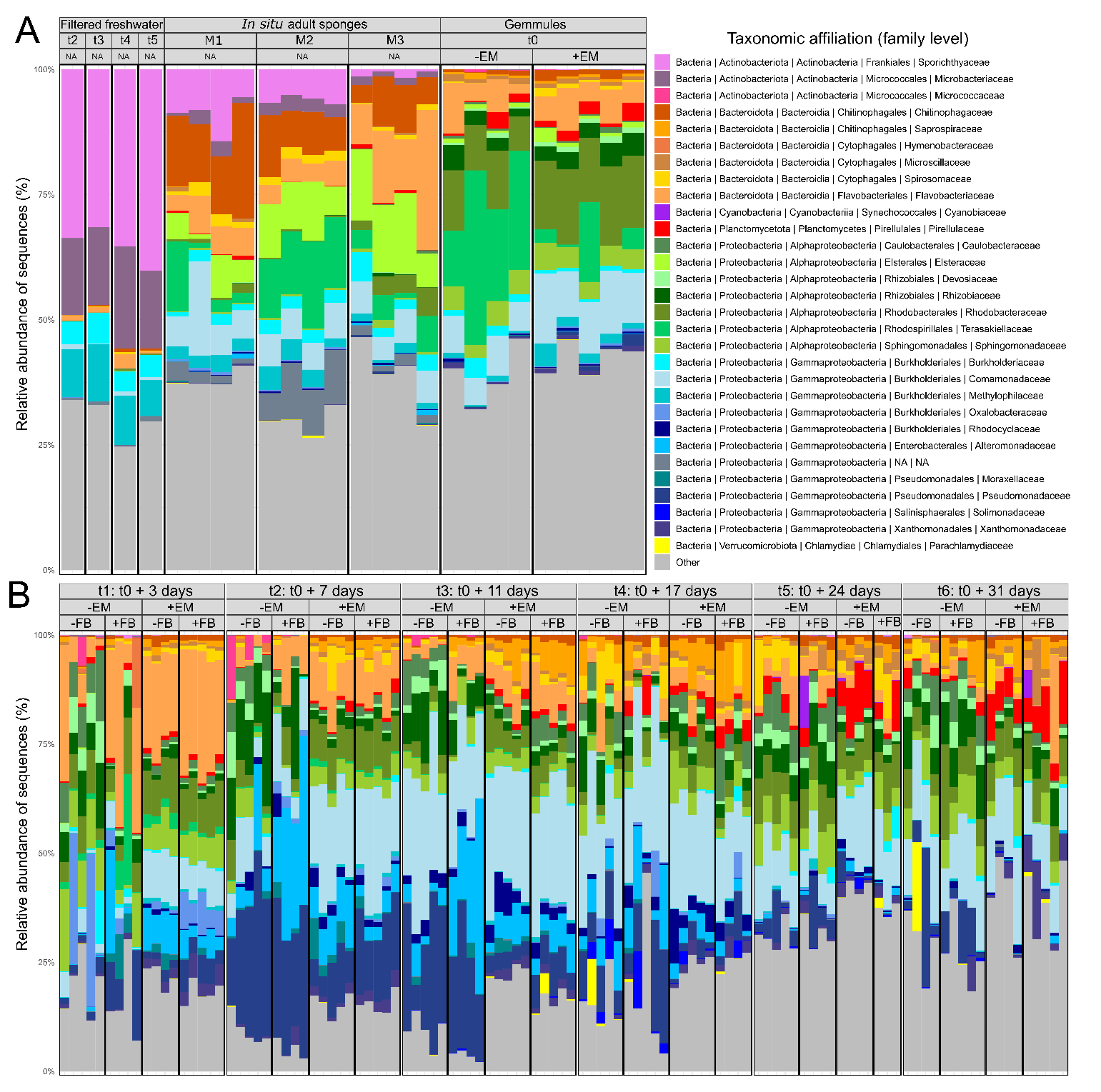


##

## Figure S7. Phylogenetical heat trees performed with gemmule samples (t_0_) and representing the taxa significantly and differentially abundant between +EM and -EM gemmules.

For each taxon, (i) the colors of their associated nodes correspond to the log2 fold change between +EM and -EM gemmules, (ii) the size of the nodes corresponds to the relative abundance of each taxa.


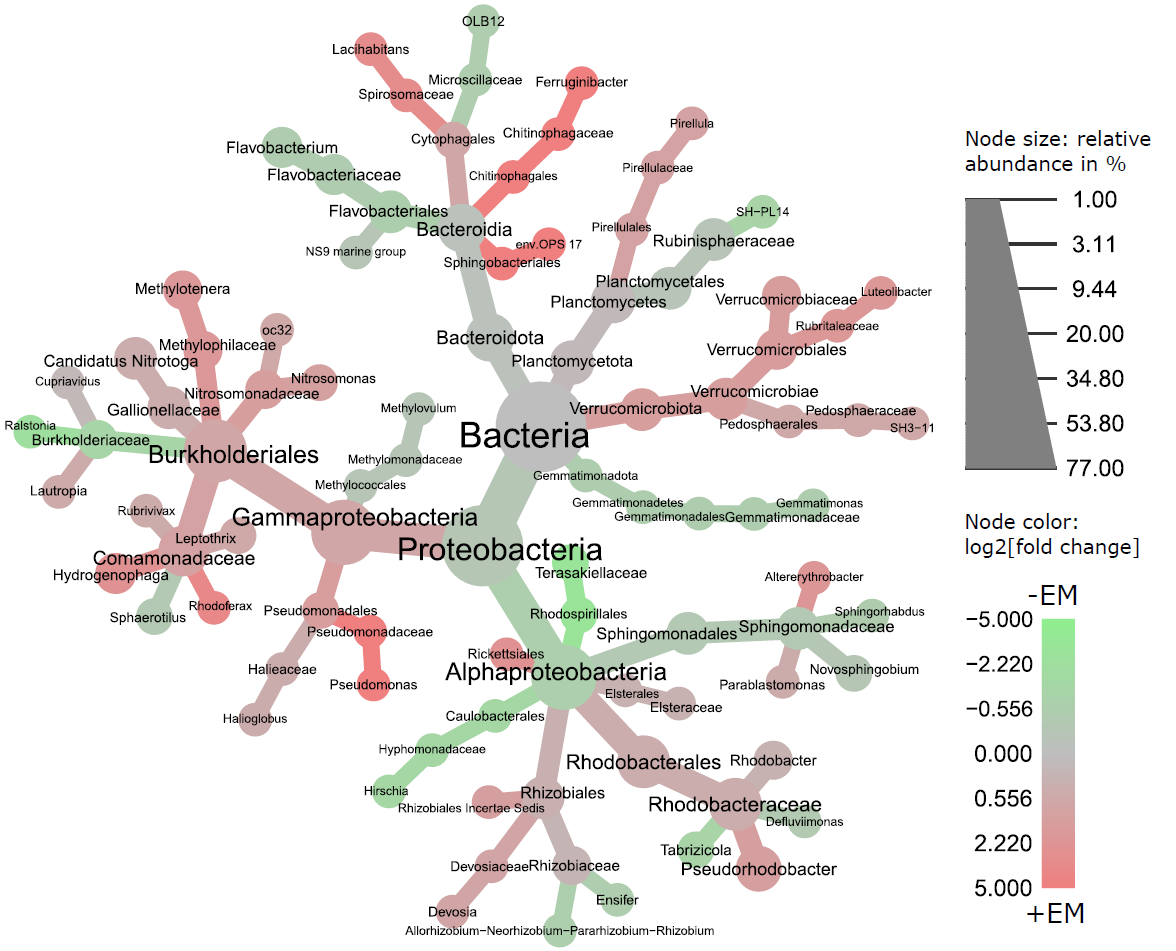


##

## Figure S8. Phylogenetical heat trees performed with juvenile sponges samples collected at t_1_ and representing the taxa significantly and differentially abundant between the +FB and -FB treatments within +EM (A) and -EM samples (B).

For each taxon, (i) the colors of their associated nodes correspond to the log2 fold change between the ratio of the mean relative abundance within each treatment, (ii) the size of the nodes corresponds to the relative abundance of each taxon. Abbreviations: EM for epibiotic microbiome, and FB for free-living bacteria.


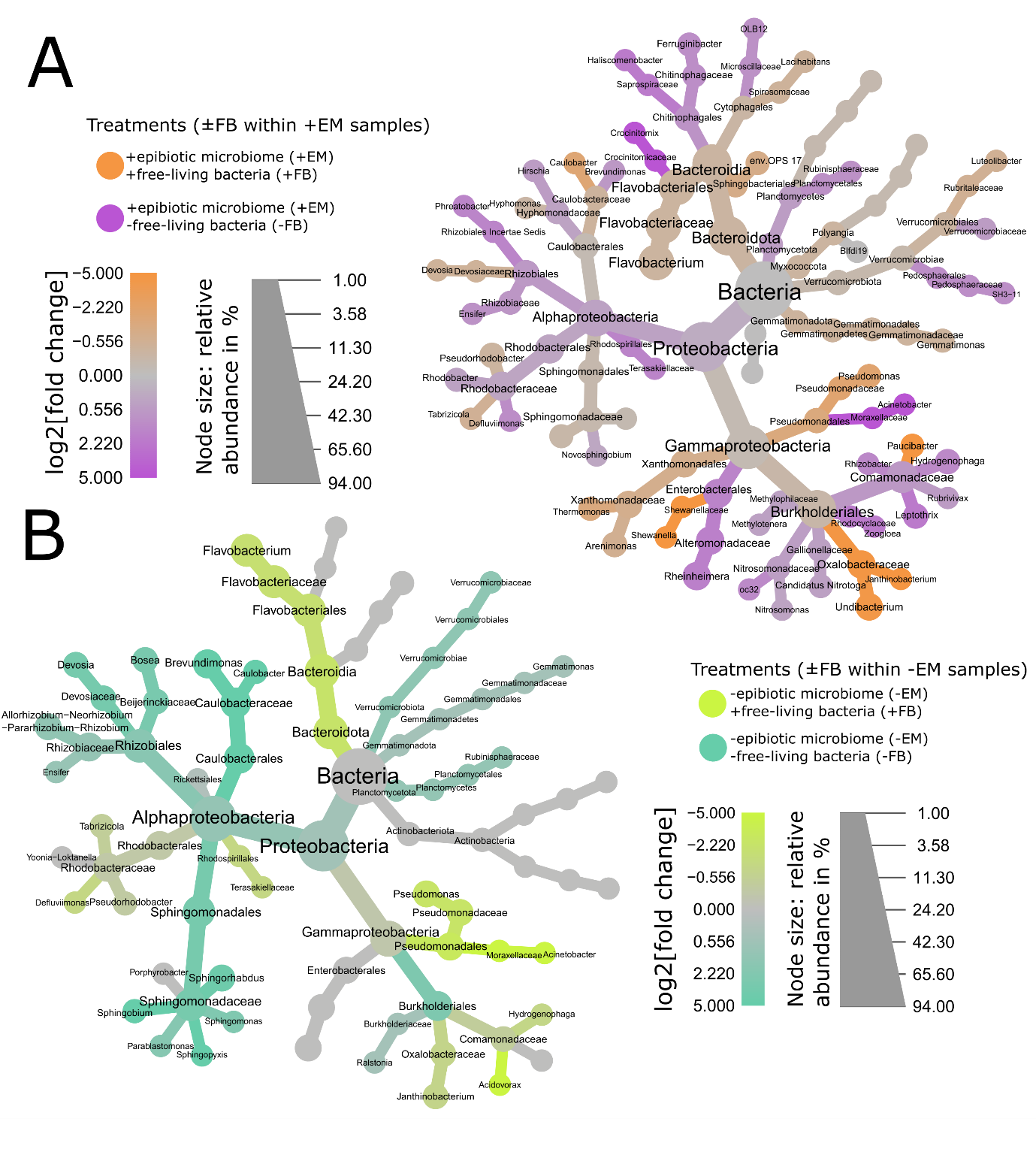


## Figure S9. Summary of the numbers of ASVs shared between the gemmules or juvenile sponges, the filtered freshwater (FF0.45), and the *in situ* adult sponges, for each sampling time.


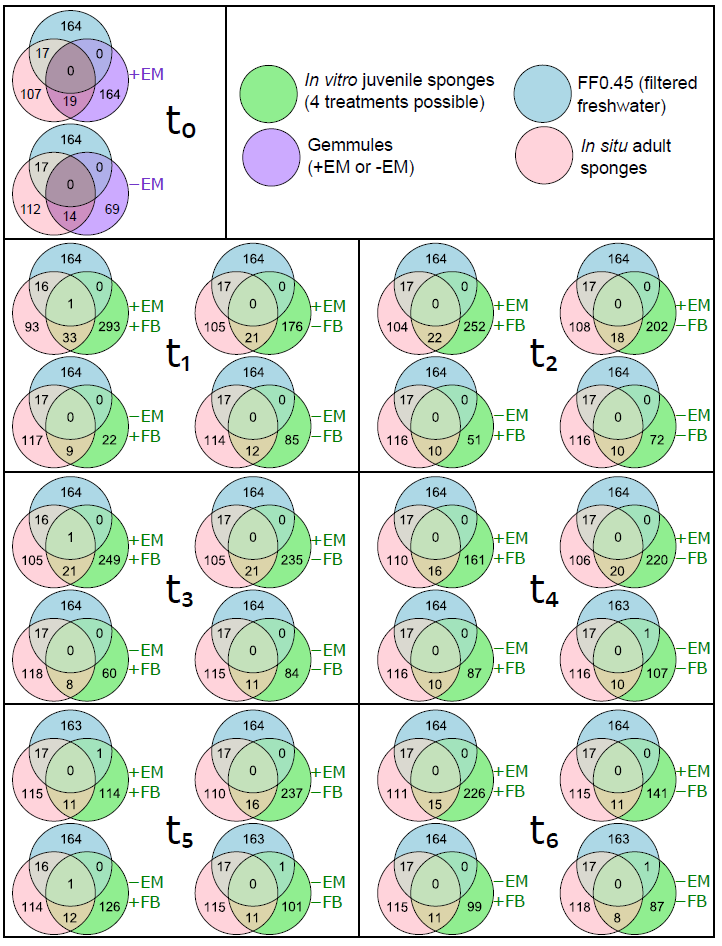


## Figure S10. Composition (relative abundances in %) of the ASVs shared between the juveniles and the *in situ* adult sponges. Taxonomical affiliation of the ASVs was indicated at the family level.

Lowercase indices (a to d) represent results from the HSD Tukey’s tests comparing each sampling time.


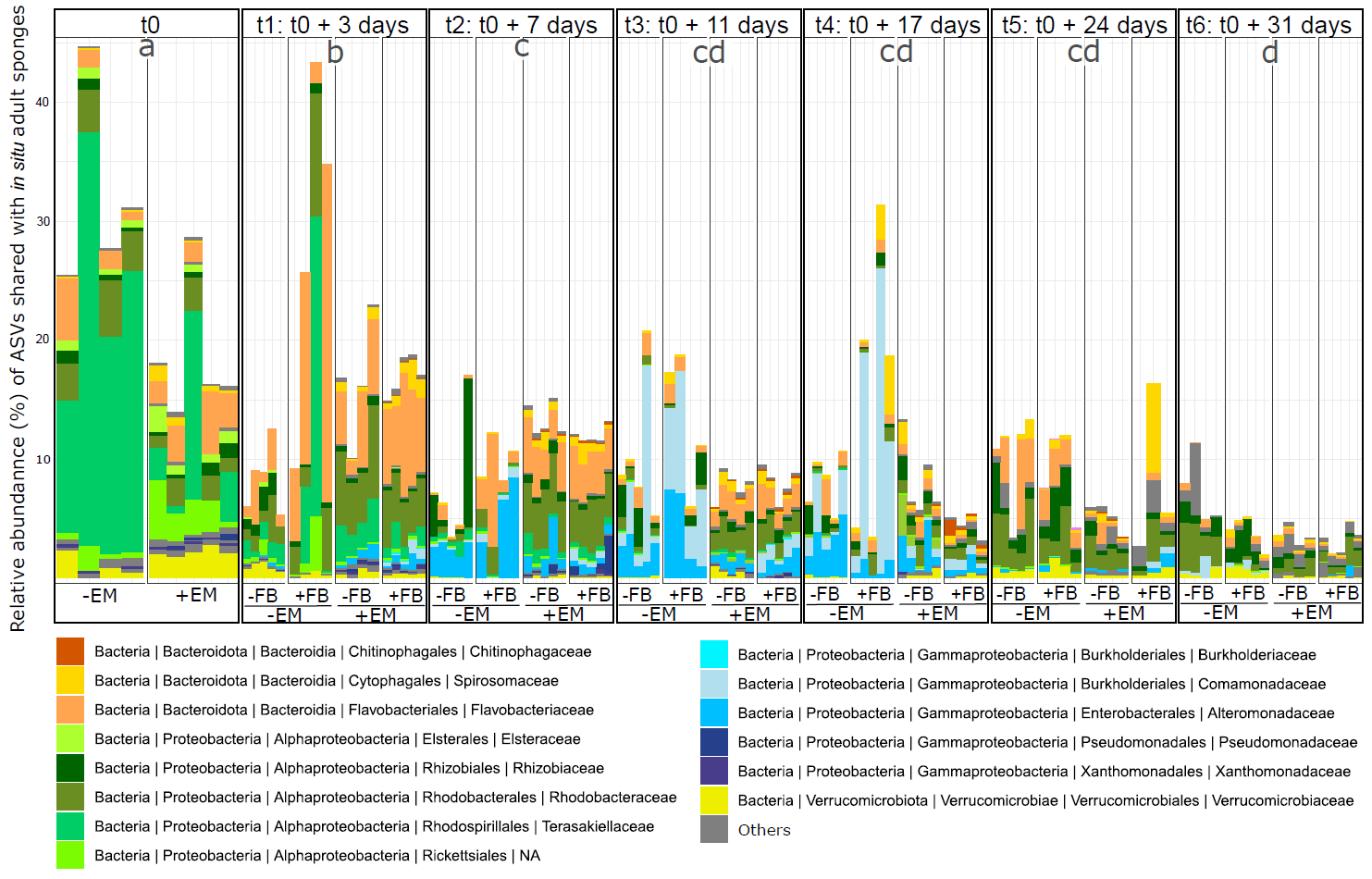

Supplement: Supplementary file 1 — Supplementary Material 1 [file 40793_2024_580_MOESM1_ESM.docx]
